# Guinea pig δβγ-ENaC is locked in an open state and uncoupled from regulation by proteases

**DOI:** 10.1007/s00424-026-03173-0

**Published:** 2026-04-25

**Authors:** Rene Yufenyuy Lawong, Etang Collins Etang, Fabian May, Philipp Vorrat, Oliver Rauh, Mike Althaus

**Affiliations:** 1 Institute for Functional Gene Analytics, Bonn-Rhein-Sieg University of Applied Sciences, Von-Liebig-Str. 20, 53359 Rheinbach, Germany; 2 Institute for Animal Physiology, Justus-Liebig-University, Giessen, Germany

**Keywords:** Epithelial Sodium Channel, ENaC, SCNN1D, Protease

## Abstract

The epithelial sodium channel (ENaC) plays a key role in salt and water homeostasis in tetrapod vertebrates. There are four ENaC subunits (α, β, γ and δ) which form heterotrimeric αβγ- or δβγ-ENaC assemblies. ENaC activity is tightly coupled to proteolytic processing of ENaC subunits, but this effect is specific to ENaC subunit assembly and mechanistically not completely understood. Guinea pig αβγ- or δβγ-ENaCs were heterologously expressed in *Xenopus* oocytes and their control by extracellular proteases was investigated using protein biochemistry, two-electrode voltage-clamp and patch-clamp electrophysiology. Guinea pig αβγ-ENaC activity was tightly coupled to cleavage of its α- and γ-subunits by the endoprotease furin and extracellular chymotrypsin or trypsin. By contrast, δβγ-ENaC activity was not affected by proteases, despite cleavage of its γ-subunit by chymotrypsin. Experiments using a β_S521C_-ENaC substitution, which locks ENaC in an open state after exposure to the sulfhydryl-reagent [2-(trimethylammonium)ethyl] methanethiosulfonate (MTSET), demonstrated that guinea pig δβ_S521C_γ-ENaCs are almost fully open when expressed in *Xenopus* oocytes. On-cell single-channel patch-clamp recordings confirmed that the open probability of guinea pig δβγ-ENaC is nearly 90%. These data indicate that guinea pig δβγ-ENaC is locked in an open state and thereby uncoupled from channel control by proteases.

**Supplementary Information:**

The online version contains supplementary material available at 10.1007/s00424-026-03173-0.

## Introduction

The epithelial sodium channel (ENaC) is a heterotrimeric, sodium-selective ion channel that plays a key role in the control of sodium homeostasis in tetrapod vertebrates. In mammals, ENaC is located in the apical membrane of sodium-absorbing epithelia such as those of the aldosterone-sensitive distal nephron, the colon, the respiratory system, salivary ducts, and the reproductive system, where it mediates the rate-limiting step of transepithelial sodium absorption [29]. Canonical ENaC is composed of the α-, β- and γ-subunits which arrange in a counterclockwise assembly [26]. The structure of each subunit resembles a clenched hand holding a ball of β-sheets. Key domains in the extracellular domain are therefore termed ‘finger’, ‘thumb’, ‘knuckle’, ‘palm’ and ‘wrist’ [26]. In the heterotrimeric assembly, the β-subunit likely serves as a structural scaffold, while the α- and γ-subunits are important for channel regulation [15].

ENaCs are constitutively active [21]. As such, numerous mechanisms regulate ENaC activity to match functional needs [19]: Glucocorticoid and mineralocorticoid hormones control ENaC subunit expression and regulate the number of channels in the membrane. ENaC membrane abundance is further controlled by kinase-regulated delivery of channels to and removal from the membrane. The open probability (P_o_) of membrane-located ENaCs is influenced by the extracellular environment, for example, sodium and chloride ions, as well as protons control ENaC P_o_ via direct interactions with the extracellular domain of the channel’s α-subunit [19]. In addition, mechanical forces such as laminar shear-stress affect its activity due to tethers with the extracellular matrix [22].

A characteristic feature is the control of ENaC function by proteases [2]. The α- and γ-subunits contain inhibitory peptides which are embedded in the ‘Gating Release of Inhibition by Proteolysis’ (GRIP) domains of the subunit’s finger regions that lock the channel in a low P_o_ state [27]. Both intra- and extracellular proteases have been shown to increase ENaC P_o_ by cutting at specific sites within the GRIP domains of the α- and γ-subunits to release their inhibitory peptides [1]. As newly synthesized ENaC translocates through the trans-Golgi network, the α-subunit is cleaved by furin, a serine protease of the proprotein convertase family, at two highly conserved RXXR$\downarrow$ (Arg-X-X-Arg$\downarrow$) consensus sites flanking the α-subunit‘s inhibitory peptide [6, 16]. The release of this inhibitory peptide raises the channels’ P_o_ from a low to a medium level. The γ-subunit, on the other hand, contains just a single RXXR$\downarrow$ furin cleavage consensus motif proximal to its inhibitory peptide and furin therefore cuts the γ-subunit only at this site [14, 16]. These furin-processed ENaCs with moderate activity dominate the ENaC population at the cell membrane [14]. There, several extracellular proteases like plasmin, trypsin, chymotrypsin or prostasin have the capacity to cleave the γ-subunit distal to the furin cleavage site [1], releasing the γ-subunits’ inhibitory peptide and strongly increasing P_o_ [8, 13]. Some of these proteases are expressed in the same cell as ENaC, thereby contributing to the control of ENaC activity. For example, proteolytic control of ENaC activity has been suggested to play an important role in airway epithelia, where a ratio of soluble protease inhibitors and membrane-anchored proteases might adjust ENaC activity to liquid volumes lining the airway surfaces [24, 35]. In the kidneys, increased K^+^ intake as well as aldosterone infusion enhance cleavage of the γ-subunit in rodents [7, 32]. Aldosterone induces the expression of channel-activating proteases such as membrane-anchored prostasin [25]. These findings demonstrate that proteolytic cleavage of the γ-subunit contributes to aldosterone-mediated increase in ENaC-activity under physiological conditions in vivo, although disruption of the γ-subunit furin cleavage site in mice has modest effects on liquid and electrolyte homeostasis [28]. In a pathophysiological scenario, excess filtration of proteases into urine in nephrotic syndrome has been suggested to cause enhanced ENaC activity and sodium retention [3].

In addition to canonical αβγ-ENaC, a fourth δ-subunit can replace the α-subunit, forming a δβγ-ENaC assembly with altered functional properties as well as different tissue distribution [15, 37]. During vertebrate evolution, the *SCNN1D* gene encoding the δ-subunit appeared with the water-to-land transition of tetrapod vertebrates [35] and disappeared in mammals returning to marine environments [38]. Furthermore, there is no functional *SCNN1D* gene in the rodent family Muridae (mice and rats), further complicating its analysis [11].

The well understood control of αβγ-ENaC by proteolytic processing is vastly different in the δβγ-ENaC counterparts. Haerteis and colleagues (2009) demonstrated that extracellular chymotrypsin could activate human δβγ-ENaC but to a lesser extent in comparison to human αβγ-ENaC. Although a furin consensus motif is present in human δ-ENaC, no furin-mediated processing of the subunit in *Xenopus* oocytes has been observed [13]. In *Xenopus laevis*, the presence of the δ-subunit completely abolished the activating effect of extracellular chymotrypsin observed for αβγ-ENaC [37]. In this orthologue, furin does not cleave the δ-subunit due to a lack of consensus cleavage sites, while furin-mediated cleavage of the γ-subunit is retained. However, while the *Xenopus* γ-subunit in the αβγ-ENaC assembly is cleaved by chymotrypsin, this subunit was not cleaved by chymotrypsin in the δβγ-ENaC assembly [37]. The presence of the δ-subunit therefore has species-specific outcomes: it either blunts the effect of extracellular chymotrypsin (as in human δβγ-ENaC) or completely abolishes it (as in *Xenopus* δβγ-ENaC).

We have previously shown that guinea pigs (*Cavia porcellus*) are rodents that have functional αβγ- and δβγ-ENaCs [11]. Like *Xenopus* ENaC orthologues, guinea pig δβγ-ENaC is not activated by extracellular proteases, however, the mechanisms underlying this observation remain unknown [11]. Here, we investigated proteolytic control of guinea pig αβγ- and δβγ-ENaCs. We show that while guinea pig αβγ-ENaC activity is tightly controlled by furin- and extracellular protease-mediated cleavage of its α- and γ-subunits, δβγ-ENaC activity is not affected by proteases. In contrast to *Xenopus* orthologues, furin can cleave the γ-subunit in the δβγ-ENaC assembly, but channel activity is uncoupled from this proteolytic processing. We demonstrate that guinea pig δβγ-ENaC is locked in an open state with an intrinsically high open probability, which is independent of proteolytic cleavage.

## Materials and methods

### cRNA synthesis and heterologous expression in *Xenopus laevis* oocytes

The DNA coding sequences of the four guinea pig ENaC subunits (α, β, γ, and δ) were present in the pTNT expression vector (Promega, Walldorf, Germany) [11]. Plasmids were used to transform NEB 5-alpha competent *Escherichia coli* (New England Biolabs, Frankfurt am Main, Germany). The plasmids were isolated using the Monarch^®^ Plasmid Miniprep kit (New England Biolabs) and cleaned using the Monarch^®^ DNA and PCR cleanup kit (New England Biolabs) according to the manufacturer‘s instructions. Cleaned plasmids were eluted in nuclease-free water and used for cRNA synthesis. cRNA was synthesised for each ENaC subunit by in vitro transcription using either the mMESSAGE mMACHINE T7 Kit (ThermoFisher Scientific, Waltham, MA, USA) or HiScribe^®^ T7 ARCA mRNA Kit (New England Biolabs) in accordance with the manufacturer’s instructions. The freshly synthesised cRNA was treated with TURBO DNase (New England Biolabs) to remove plasmid DNA and then cleaned using the MEGAclear Transcription Clean-up Kit (ThermoFisher Scientific) or the Monarch^®^ RNA cleanup kit (New England Biolabs) according to the manufacturer‘s instructions and eluted in nuclease-free water. The cRNAs were then combined into αβγ- or δβγ-ENaC subunit combinations in concentrations of 5 ng/µl per ENaC subunit for electrophysiological recordings, or 300 ng/µl per ENaC subunit for immunoblotting. cRNA was stored at −80 °C.

Stage V/VI *Xenopus laevis* oocytes were purchased from Ecocyte Bioscience (Dortmund, Germany) and stored in Modified Barth’s Solution (MBS) containing (in mM): 88 NaCl, 1 KCl, 2.4 NaHCO_3_, 0.82 MgSO_4_, 0.33 Ca(NO_3_)_2_, 0.41 CaCl_2_ and 10 HEPES (4-(2-hydoxyethyl)−1-piperazineethanesulfonic acid), at pH = 7.5 adjusted with NaOH, and supplemented with 20 µg/ml gentamycin (ThermoFisher Scientific) at 4 °C until use. For heterologous ENaC expression, 18.4 nl of the combined αβγ- or δβγ-ENaC cRNAs were injected into oocytes using a Nanoject II microinjector (Drummond Scientific Company, Broomall, PA, USA). This corresponded to a total cRNA amount of 0.09 ng per ENaC subunit per oocyte for TEVC recordings and 5.5 ng per ENaC subunit per oocyte for biochemical experiments. Injected oocytes were stored at 16 °C in N-methyl-D-glucamine (NMDG) Oocyte Ringer Solution containing (in mM): 10 NaCl, 1 KCl, 2 CaCl_2_, 2.5 Na^+^-pyruvate, 80 NMDG, 5 HEPES at pH = 7.4 adjusted with HCl and supplemented with 20 µg/ml gentamycin.

### Epitope tagging of ENaC subunits

For immunodetection, the guinea pig α-, δ-, and γ-ENaC subunits were coupled with an N-terminal HA-tag and a C-terminal V5-tag using cloning primers listed in Table 1. Fragments were amplified by PCR using Q5^®^ High-Fidelity DNA polymerase (New England Biolabs). An initial denaturation step was done at 98 °C for 30 s, followed by 30 cycles of (i) denaturation at 98 °C for 10 s, (ii) annealing at 61 °C for 30 s, and (iii) elongation at 72 °C for 150 s. A final elongation was done at 72 °C for 2 min and the amplicons were separated and visualized on a 1% agarose gel stained with Midori Green Advance (Nippon Genetics Europe, Düren, Germany) as per the manufacturer’s instructions. Amplicons were purified from the agarose gels using the Monarch^®^ Spin DNA Gel Extraction Kit (New England Biolabs) according to the manufacturer’s instructions. The purified amplicons were ligated into the pJET1.2 plasmid vector (CloneJET PCR Cloning Kit, ThermoFisher Scientific) according to the manufacturer’s instructions. The plasmids were then used to transform competent NEB 5-alpha *E. coli* (New England Biolabs), isolated and cut with fast digest restriction enzymes, XhoΙ, EcoRΙ, KpnΙ and NotΙ (ThermoFisher Scientific). After gel purification as described above, cleavage products were ligated into the pTNT expression vector (Promega) using the Quick Ligation™ Kit (New England Biolabs) according to the manufacturer’s instructions. The plasmids were then used to transform competent NEB 5-alpha *E. coli* (New England Biolabs) and then later isolated as described above. Successful epitope tagging was verified by Sanger sequencing (Eurofins Genomics, Ebersberg, Germany).

**Table 1:** Sequences of primers employed for HA- and V5-epitope tagging of guinea pig ENaC subunits. The cleavage sites for restriction enzymes are shown in lower case letters, the Kozac sequences are underlined, the HA- and V5-epitopes are highlighted in bold, and the ENaC overlapping sequences in italics

Primer		Sequence (5’—3’)
_HA_α_V5_-ENaC	Forward	GTTCTctcgagGCCACCATG**TACCCATACGATGTTCCAGATTACGCT***AAAGGCGATGAATTGAAAGCTCAG*
	Reverse	TTTggtaccTCA**CGTAGAATCGAGACCGAGGAGAGGGTTAGGGATAGGCTTACC***GGGTTCCCTGGGAGCACATGCGGCAC*
_HA_γ_V5_-ENaC	Forward	TTCTTgaattcGCCACCATG**TACCCATACGATGTTCCAGATTACGCT***GCTCCCGGAGAGAAGATC*
	Reverse	GTTggtaccTCA**CGTAGAATCGAGACCGAGGAGAGGGTTAGGGATAGGCTTACC***AGGCTCACTGGGCACCTGAGTATC*
_HA_δ_V5_-ENaC	Forward	GTTCTgaattcGCCACCATG**TACCCATACGATGTTCCAGATTACGCT***CCTCCTTTGGAAAGAGGCCAC*
	Reverse	TTTgcggccgcTCA**CGTAGAATCGAGACCGAGGAGAGGGTTAGGGATAGGCTTACC***TCCCAGCTCTTCCATAAGTAC*

### Site-directed mutagenesis

The Q5^®^ site-directed mutagenesis kit (New England Biolabs) was used to perform site-directed mutagenesis reactions as per the manufacturer’s instructions. Plasmids containing guinea pig β-ENaC or HA- and V5-tagged α- and γ-ENaCs were used as templates. Mutagenesis primers are listed in Table 2. In order to obtain the furin-insensitive α-ENaC constructs, primers mutating the distal furin cleavage site, α_R198Q,R199Q,A200Q,R201Q_, were used on α_R175Q,S176Q,R177Q,R178Q_ constructs where the proximal furin cleavage site was already mutated. After mutagenesis PCR, plasmids were treated with KLD enzyme mix in order to circularise the amplification products and to digest the template DNA. Plasmids were cleaned with either the MEGAclear Transcription Clean-up Kit (ThermoFisher Scientific) or the Monarch^®^ PCR and DNA cleanup kit (New England Biolabs) according to the manufacturer ‘s instructions, subsequently used to transform competent NEB 5-alpha *E. coli* and plasmids were isolated as described above. Successful mutagenesis was verified by Sanger sequencing (Eurofins Genomics).

**Table 2:** Sequences of primers employed for site-directed mutagenesis. Nucleotide exchanges for site-directed mutagenesis are highlighted in lower case letters

Primer		Sequence (5’—3’)
β_S521C_	Forward	TGGCTCCTCTgcAATCTGGGTG
	Reverse	GACGATATTATTGGCTGC
γ_R136Q,K137Q,R138Q,R139Q_	Forward	cagcagGACACCGAGTCTGAAAATC
	Reverse	ctgctgAGACGTAACCTCTGAAAAG
α_R175Q,S176Q,R177Q,R178Q_	Forward	cagcagAGCCTTGCTGATACACTC
	Reverse	ctgctgTGCTCCAGCCAGCAAAG
α_R198Q,R199Q,A200Q,R201Q_	Forward	cagcagTCTTCAGACCCTAGTTC
	Reverse	ctgctgGGGTTCAGGCTGAAC

### Replacement of the knuckle domain by NEBuilder^®^ HiFi DNA assembly

Plasmids containing the guinea pig δ-subunit with its knuckle domain replaced with that from the guinea pig α-subunit were generated using the NEBuilder^®^ HiFi DNA Assembly kit (New England Biolabs) following the manufacturer’s instructions. With PCR, the plasmid containing the δ-subunit was linearized using the linearization primers listed in Table 3 such that the knuckle domain of the δ-subunit was deleted. The knuckle domain of the α-subunit was then amplified using primers that had overhangs homologous to the sequence of the δ-subunit. The PCRs were carried out using the Q5^®^ High-Fidelity DNA polymerase (New England Biolabs). The two PCR conditions had an initial denaturation step at 98 °C for 30 s, followed by 35 cycles of (i) denaturation at 98 °C for 10 s, (ii) annealing at 58 °C for 30 s, and (iii) elongation at 72 °C for 150 s. A final elongation was done at 72 °C for 2 min and the amplicons were separated and visualized on a 1% agarose gel stained with Midori Green Advance (Nippon Genetics Europe, Düren, Germany) as per the manufacturer’s instructions. Amplicons were purified from the agarose gels using the Monarch^®^ Spin DNA Gel Extraction Kit (New England Biolabs) according to the manufacturer’s instructions. The two PCR products were then assembled with the NEBuilder^®^ HiFi DNA Assembly master mix by incubating the fragments in the master mix at 50 °C for 60 min. The assembly products were then used to transform competent NEB 5-alpha *E. coli* and plasmids were isolated as described above. Successful assembly was verified by Sanger sequencing (Eurofins Genomics).

**Table 3:** Sequences of primers employed for NEBuilder® HiFi DNA Assembly. Nucleotide overhangs homologous to the δ-subunit are underlined while the nucleotides homologues to the α-subunit knuckle are highlighted in bold letters

Primer		Sequence (5’—3’)
Linearisation of the plasmid containing the δ-subunit	Forward	GCAAAAGTGAATATCTTCTACCAGGAGC
	Reverse	AGATTTGGCTGAAGGCCAACGAC
Amplification of the knuckle domain of the α-subunit	Forward	GTCGTTGGCCTTCAGCCAAATCT**CAGGACTGGATTTTTCAGATGCTGTC**
	Reverse	GCTCCTGGTAGAAGATATTCACTTTTGC**GACCCCATTTCTTTTATTTGAAATTGTGTAATTG**

### Immunodetection of HA- and V5-tagged ENaC proteins

Western blot analyses were performed on lysates from *Xenopus* oocytes expressing HA- and V5-tagged ENaC proteins. First, from every set of oocytes injected with a particular ENaC-subunit combination, expression was confirmed when amiloride-sensitive currents could be detected from 2-3 of the injected oocytes using two-electrode voltage-clamp (TEVC) recordings. Using a 1000 µl pipette and then a 26-gauge needle, the oocytes were homogenized in 10 µl/oocyte of oocyte lysis buffer containing (in mM): 83 NaCl, 10 HEPES, 1 MgCl_2_ and 1% Triton X-100 at pH = 7.4 and supplemented with protease inhibitors (cOmplete™ Mini, EDTA-free protease inhibitor cocktail tablets, Roche Diagnostics, Mannheim, Germany). The lysates were then centrifuged at 1,500 × g for 10 min at 4 °C and the aqueous fraction was then collected while avoiding contamination with the fatty layer. This procedure was repeated 2-3 times. 7.5 µl of 4X SDS protein loading buffer (Roti^®^-load 1, Carl Roth, Karlsruhe, Germany) or 4X Laemmli Sample Buffer supplemented with 2-mercaptoethanol (Bio-Rad Laboratories, Munich, Germany) was then added to 22.5 µl of the samples. Samples were heated at 95 °C and 5-25 µl were separated by sodium dodecyl sulfate polyacrylamide gel electrophoresis (SDS-PAGE) on either 4–20% gradient or 10% SDS polyacrylamide gels. The separated proteins were then transferred onto polyvinylidine difluoride (PVDF) membranes using either the wet blotting method with the Mini Trans-Blot cell (Bio-Rad Laboratories) or the semi dry blotting method with the Trans-Blot Turbo Transfer System (Bio-Rad Laboratories) according to the manufacturer’s instructions. The membranes were then blocked at room temperature for 60 min in TBST (150 mM NaCl, 15 mM Tris–HCl, 4.6 mM Tris-base, and 0.1% Tween 20) with 5% skim milk (Carl Roth) under constant rocking. The membranes were then incubated overnight with constant rocking at 4 °C in TBST/5% skim milk containing a 1:10,000 dilution of monoclonal mouse IgG anti-HA (Invitrogen, Rockford II, USA) or a 1:5,000 dilution of monoclonal mouse IgG2b anti-V5 (BioLegend, San Diego, USA) primary antibodies. The membranes were then washed 3 × 10 min in TBST and then incubated for one hour at room temperature in a 1:5,000 dilution of a peroxidase-conjugated rabbit anti-mouse IgG secondary antibody (Invitrogen) in the blocking buffer. The membranes were again washed 3 × 10 min in TBST. Bands were detected using a chemiluminescence solution (1 ml of solution A (200 ml of 0.1 M Tris–HCl and 50 mg of Luminol), 100 µl of solution B (11 mg of para-hydroxycoumaric acid dissolved in 10 ml DMSO) and 0.3 µl of 30% H_2_O_2_) and images were captured using the ChemiDoc™XRS + imager (Bio-Rad Laboratories).

### Detection of cell surface ENaC fractions by biotinylation and streptavidin-pulldown

First, from every set of oocytes injected with a particular ENaC-subunit combination, expression was confirmed when amiloride-sensitive currents could be detected from 2-3 of the injected oocytes by TEVC recordings. To minimize the possibility of active internalization of biotin into the cells, all biotinylation steps were carried out on ice. 20–30 oocytes per experimental group were washed in chilled buffer A (90 mM NaCl, 5 mM triethanolamine, 3 mM KCl, 1 mM CaCl_2_ at pH = 8.0) and then incubated with continuous rocking for 20 min in buffer A supplemented with 1 mg/ml of EZ-Link™ Sulfo-NHS-SS-Biotin (ThermoFisher Scientific). The oocytes were then incubated for 10 min in buffer A supplemented with 50 mM glycine to quench and remove excess biotin. Using a 1000 µl pipette tip and then a 27-gauge needle, the oocytes were lysed in 1 ml of lysis buffer (90 mM NaCl, 20 mM Tris, and 1% Triton X-100, pH = 7.4) supplemented with protease inhibitors (cOmplete™ Mini, Roche Diagnostics). The lysates were vortexed for 10–20 s and then incubated on ice for 15 min. Following incubation, the lysates were centrifuged for 10 min at 8,000 × g at 4 °C. The aqueous phase of the supernatant was then collected carefully, avoiding the fatty layer on top. To ensure complete avoidance of the fatty layer, the process was repeated 3–4 times. 100 µl of Pierce™ NeutrAvidin™ agarose resin (ThermoFisher Scientific) were washed in 500 µl of the lysis buffer and then centrifuged at 1,500 × g for 3 min at 4 °C. The aqueous lysates were then added onto the cleaned resin and incubated overnight at 4 °C with gentle continuous shaking. Samples were then centrifuged at 1,500 × g for 3 min at 4 °C so the resin with the biotin-bound membrane proteins could sediment to the bottom, leaving the non-biotinylated cytosolic protein fractions in the aqueous supernatant. The cytosolic fractions were then collected while the resin with membrane proteins were washed three times with lysis buffer at 1,500 × g, for 3 min at 4 °C and three times in lysis buffer supplemented with 300 mM NaCl at 1,500 × g, for 3 min at 4 °C. In order to dissociate the avidin–biotin complexes and free the membrane protein fractions, 100 µl of 2 × Laemmli Sample Buffer supplemented with 2-mercaptoethanol (Bio-Rad Laboratories) prepared in the oocyte lysis buffer were added to the resin and heated at 95 °C for 5 min. This also denatured the proteins, making them ready for separation by SDS-PAGE. 15–30 µl of the samples were loaded onto the gels and immunoblots were performed as described above. Evaluation of the separation of membranes from cytosolic fractions was done by reprobing the membranes overnight at 4 °C in a 1:2,000 dilution of anti β-actin mouse monoclonal IgG antibody (Santa Cruz biotechnology, Heidelberg, Germany). The membranes were then washed three times for 10 min in TBST and then incubated for one hour at room temperature in a 1:5,000 dilution of a peroxidase-conjugated rabbit anti-mouse IgG secondary antibody (Invitrogen). The membranes were again washed three times for 10 min each in TBST and β-actin was detected using an enhanced chemiluminescence solution and images captured using the ChemiDoc™XRS + imager (Bio-Rad Laboratories). Absence of β-actin bands in the membrane fractions indicated proper separation from cytosolic fractions.

### Two-electrode voltage-clamp (TEVC) recordings

Transmembrane currents of ENaC-expressing oocytes were recorded using the Roboocyte2 (Multichannelsystems, Reutlingen, Germany) automated TEVC system at room temperature as previously described [23]. Microelectrodes were prepared from borosilicate glass capillaries (World Precision Instruments, Hitchin, UK; TW150-4) using a DMZ Universal Puller (Zeitz, München, Germany) and then filled with 3 M KCl. Oocytes were placed in a 96-well plate filled with a low (1 mM) sodium-containing oocyte ringer solution (low sodium ORS) (1 mM NaCl, 1 mM KCl, 2 mM CaCl_2_, 5 mM HEPES, 89 mM NMDG and pH = 7.4 adjusted with HCl) and impaled with the capillaries containing the internal electrodes. While still in this buffer, the oocytes were clamped at a holding potential of −60 mV and recordings were performed with constant perfusion of oocyte ringer solution (ORS) (90 mM NaCl, 1 mM KCl, 2 mM CaCl_2_, 5 mM HEPES, and pH = 7.4 adjusted with NaOH) at 5 ml per minute perfusion speed. The ENaC inhibitor amiloride (100 µM) was routinely employed to determine ENaC-mediated amiloride-sensitive current fractions. Transmembrane current signals (I_M_) were recorded at a 20 Hz sampling rate.

### Patch-clamp recordings

On-cell patch-clamp recordings were performed on oocytes expressing αβγ- or δβγ-ENaC. Upon mechanical removal of the vitelline layer, oocytes were placed in bath solution (145 mM KCl, 1.8 mM CaCl_2_, 2 mM MgCl_2_, 5.5 mM glucose and 10 mM HEPES and pH = 7.4 adjusted with KOH). Borosilicate glass capillaries (TW150-4, World Precision Instruments) were coated with Sigmacote (Sigma Aldrich, Darmstadt, Germany) and pulled to fire-polished patch-pipettes (2–3 MΩ resistance) using a DMZ Universal Puller (Zeitz). In some cases, pipettes were baked for 30 min at 70 °C to enhance sealing. Patch-Pipettes were filled with pipette solution containing 145 mM NaCl, 1.8 mM CaCl_2_, 2 mM MgCl_2_, 5.5 mM glucose and 10 mM HEPES and pH = 7.4 adjusted with NaOH. Current signals were amplified using a LM-PC patch-clamp amplifier (List-Medical, Darmstadt, Germany), filtered at 1 kHz and sampled with 10 kHz using a LIH 8 + 8 AD/DA interface (HEKA Elektronik, Stuttgart, Germany) and digitally filtered at 0.1 kHz with HEKA Patchmaster 2 × 92 (HEKA). Recordings were performed at room temperature.

Current traces showing single-channel fluctuations were used to construct amplitude histograms with a bin width of 0.01 pA. To determine the occupation probabilities of the different current levels, the amplitude histograms were fitted with a sum of Gaussian functions:

1 $$f\left(x\right)\mathrm{=}{\sum }_{k\mathrm{=}0}^{N}\frac{{A}_{k}}{\sqrt{2\pi }\cdot {\sigma }_{k}}\cdot {e}^{-\frac{1}{2}\cdot \frac{\left(x-{\mu }_{k}\right)}{{\sigma }_{k}^{2}}}$$

with *f* (*x*) being the number of points in the bin with current amplitude *x*, *μ*_*k*_ the mean, *σ*_*k*_ the standard deviation, and *A*_*k*_ the total number of points belonging to the *k*-th gaussian component (*k* = 0,1,2,…, N). *N* is the minimum number of ENaC channels that explains the occurrence of the (*N* + 1) observable current levels. It was assumed that all ENaC channels in the membrane patch had the same single-channel amplitude. Hence, the mean value *µ*_*k*_ of the *k*-th component was calculated as an integer multiple of the single-channel amplitude *i*:

2 $${\mu }_{k}\mathrm{=}{\mu }_{0}\mathrm{+}k\cdot i$$

with *µ*_*0*_ being the mean of the baseline level (*k* = 0, no channel open). The occupation probabilities *P*_*k*_ were then calculated as follows:

3 $${P}_{k}\mathrm{=}{A}_{k}\cdot {\left({\sum }_{j\mathrm{=}0}^{N}{A}_{j}\right)}^{-1}$$

with *A*_*j*_ being the total number of points belonging to the *j*-th gaussian component (*j* = 0,1,2,…, N). It was assumed that all ENaC channels in a membrane patch have the same open probability. The single-channel open probability *P*_*o*_ was then determined by fitting the calculated occupation probabilities *P*_*k*_ to a binomial distribution:

4 $$B\left(k\left|{P}_{o}\right.,N\right)\mathrm{=}{{P}_{o}}^{k}\cdot {\left(1-{P}_{o}\right)}^{N-k}\cdot \left(\begin{array}{c}N\\ k\end{array}\right)$$

The analysis was performed using a customized analysis routine in Matlab R2024b (Mathworks Inc., Natick, MA, USA).

### Statistical analyses

Data for all our experimental approaches was collected from at least two separate oocyte donors and *n* represents the number of experimental repeats. Data is presented as mean ± SD. TEVC recordings were analysed using the Roboocyte 2 + software (Multichannelsystems). All statistical analyses were performed using GraphPad Prism version 10.6.0 (GraphPad Software, Boston, MA, USA). Normal distribution of data was assessed using the D’Agostino and Pearson test. Statistical significance was assessed using appropriate tests as indicated in the figure legends and statistical significance was accepted when *p* < 0.05.

## Results

### Activity of guinea pig δβγ-ENaC is not affected by extracellular proteases

**Fig. 1:**
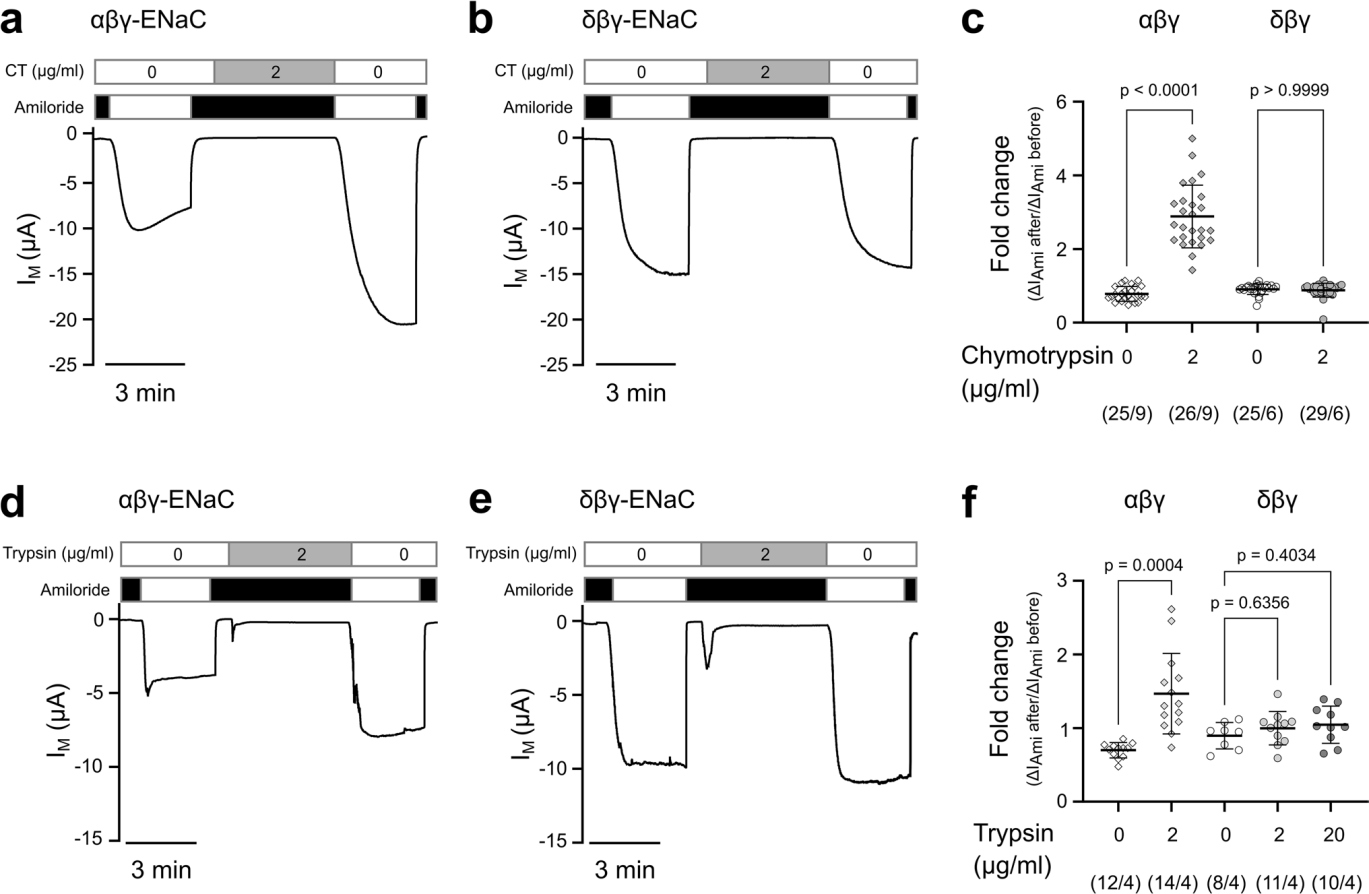
Activity of guinea pig δβγ-ENaC is not affected by extracellular proteases. (**a, b**) Representative transmembrane current (I_M_) traces obtained from TEVC recordings of oocytes expressing guinea pig αβγ-ENaC (**a**) and δβγ-ENaC (**b**) with V_M_ = −60 mV. Amiloride (100 µM, black bars) was used to determine ENaC-mediated amiloride-sensitive current fractions (ΔI_Ami_). Oocytes were incubated for 5 min in chymotrypsin (2 µg/ml, CT) in the presence of amiloride, and the impact of the protease on ΔI_Ami_ after the application of chymotrypsin was recorded. (**c**) Comparison of the fold change in ΔI_Ami_ between the chymotrypsin-treated experimental groups as shown in panels (a/b) and control groups without application of chymotrypsin. Statistical analyses were performed with Kruskal–Wallis test followed by Dunn’s multiple-comparison test. (**d-f**) Similar experiments as shown in panels (a-c), employing the protease trypsin. Comparison of the fold change in ΔI_Ami_ between the trypsin-treated experimental group and control groups without trypsin. Statistical analyses were performed with Welch and Brown-Forsythe ANOVA tests and then Dunnett’s T3 multiple-comparison test. Data points in the scatter plots are derived from *n* individual experiments. Lines and error bars indicate the mean and standard deviation of the mean. Absolute values of ΔI_Ami_ before and after protease (or mock experiments) are provided in Supplementary Figure 1. Numbers in parentheses indicate oocytes/oocyte donors

The impact of chymotrypsin was evaluated on guinea pig αβγ- or δβγ-ENaCs expressed in *Xenopus laevis* oocytes following a previously described protocol [11, 37]. We measured the amiloride-sensitive fraction of the transmembrane current (ΔI_Ami_) before and after 5 min exposure of the oocytes to 2 µg/ml chymotrypsin (Sigma Aldrich/Carl Roth) (Fig. 1a,b) and determined ENaC activity after protease treatment as the fold change in ΔI_Ami_. Amiloride was also present during the 5-min protease exposure to avoid run-down in transmembrane currents due to the accumulation of Na^+^ in the cell over time. Control experiments were performed by following the identical protocol, except that the 5 min of incubation were done without chymotrypsin. Fold changes in ΔI_Ami_ from experiments with chymotrypsin were then compared to the fold changes from the control experiments (Fig. 1c). In agreement with previously published work [11], guinea pig αβγ-ENaCs were consistently activated by chymotrypsin while the δβγ-ENaCs were not. In αβγ-ENaC expressing oocytes, the ΔI_Ami_ fold change in control experiments of 0.78 ± 0.2 (*n* = 25) was significantly increased upon chymotrypsin treatment to 2.88 ± 0.85 (*n* = 26; *p* < 0.0001). Oocytes expressing δβγ-ENaC showed a fold change of 0.90 ± 0.14 (*n* = 25) under control conditions, which was not statistically different from the fold change of 0.92 ± 0.12 (*n* = 29; *p* > 0.9999) following treatment with chymotrypsin.

Chymotrypsin cleaves the human γ-subunit after phenylalanines F_174_ and F_175_ [12]. This putative cleavage site is conserved in the guinea pig ENaC γ-subunit (F_176_ and F_177_). To rule out that the lack of proteolytic activation of δβγ-ENaC is due to limited accessibility of the γ-subunit’s cleavage site to this protease in the guinea pig ENaC orthologue, chymotrypsin was replaced with trypsin (2 µg/ml; Sigma Aldrich) and identical protocols as above were performed (Fig. 1d,e). Similar to the results obtained using chymotrypsin, the ΔI_Ami_ fold change in control experiments on guinea pig αβγ-ENaC expressing oocytes was 0.70 ± 0.10 (*n* = 12) and significantly increased to 1.47 ± 0.55 (*n* = 14; *p* = 0.0004) after exposure to trypsin (Fig. 1f). For oocytes expressing δβγ-ENaC, there was no significant difference between the ΔI_Ami_ fold change in control oocytes (0.90 ± 0.18; *n* = 8) and the ΔI_Ami_ fold change in oocytes treated with trypsin (1.00 ± 0.23; *n* = 11; *p* = 0.6356) (Fig. 1f). To rule out that this lack of activation was a matter of sensitivity, the concentration of trypsin was increased tenfold (20 µg/ml). Identical experiments did not change the ΔI_Ami_ fold change of guinea pig δβγ-ENaC (1.05 ± 0.25; *n* = 10; *p* = 0.4034) (Fig. 1f). Taken together, these data demonstrate that guinea pig αβγ-ENaC is activated by proteases, whereas δβγ-ENaC is not.

### Guinea ENaC isoforms show distinct subunit cleavage patterns

**Fig. 2:**
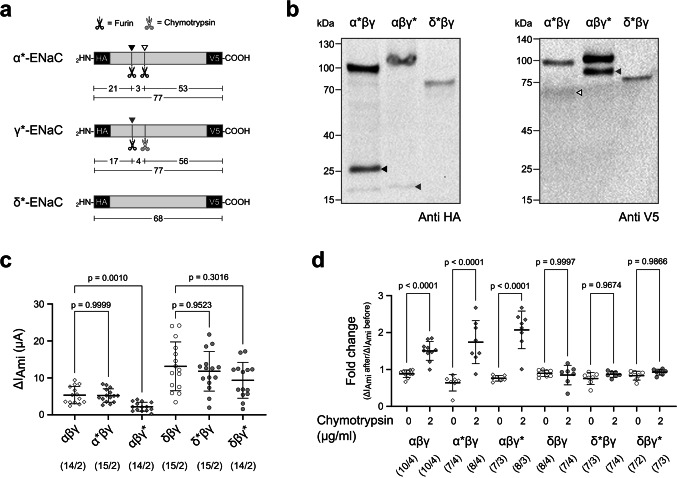
Guinea ENaC subunits show distinct cleavage patterns. (**a**) Schematic depiction of epitope-tagged ENaC subunits (grey bars) containing N-terminal HA- and C-terminal V5-tags (black bars). Numbers indicate the molecular mass (kDa) of the peptide fragments, while the scissors indicate where proteases cleave the subunits. Asterisks indicate the presence of both HA- and V5 tags in a subunit. (**b**) Immunoblots using anti-HA and anti-V5 antibodies on whole-cell lysates from oocytes expressing α*βγ-ENaC, αβγ*ENaC, or δ*βγ-ENaC. Glycosylation of the ENaC subunits is the likely reason why the observed molecular sizes are larger than indicated in panel (a). The α*-subunit shows two bands corresponding to full-length and furin-cleaved fragments. The γ*-subunit also shows two bands corresponding to full-length and furin-cleaved fragments. The δ*-subunit shows just a single band, consistent with the lack of furin consensus sites in this subunit. Filled and unfilled black arrowheads indicate fragments due to proximal and distal furin-mediated cleavage in the α*-subunit as shown by the same arrowheads in (a). Filled grey arrowheads indicate fragments due to furin-mediated cleavage in the γ*-subunit as shown by the same arrowhead in (a). The blots represent one of *n* = 2–3 independent experimental repeats. Original uncropped versions of the gels and blot images shown in this panel are provided in Supplementary Figure 2. (**c**) Statistical comparison of the amiloride-sensitive current fractions (ΔI_Ami_) derived from TEVC recordings of oocytes expressing untagged ENaCs with oocytes expressing ENaCs with one of the subunits containing the epitope tags (*), V_M_ = −60 mV. Statistical analyses were performed with Welch and Brown-Forsythe ANOVA tests and then Dunnett’s T3 multiple-comparison tests. (**d**) The impact of epitope-tagging on the response of the ENaC assemblies to chymotrypsin. Data is derived from TEVC experiments as shown in Fig. 1, panels (a-c). Statistical analyses were performed with Ordinary one-way ANOVA and then Sidak’s multiple-comparison test. Data points in the scatter plots are derived from *n* individual experiments. Lines and error bars indicate the mean and standard deviation of the mean. Absolute values of ΔI_Ami_ before and after protease (or mock experiments) are provided in Supplementary Figure 3. Numbers in parentheses indicate oocytes/oocyte donors

To evaluate the proteolytic processing of guinea pig αβγ- and δβγ-ENaC by immunoblotting, the α-, δ-, and γ-subunits were tagged with an N-terminal HA-tag and a C-terminal V5-tag. The presence of both tags is indicated with an asterisk symbol following the subunit’s name (Fig. 2a). The β-subunit was not tagged because it is not proteolytically processed. The α*βγ-ENaC, αβγ*-ENaC or δ*βγ-ENaC subunit combinations were expressed in *Xenopus* oocytes.

Immunoblots targeting the N-terminal HA-tag in the α*-subunit revealed a band at ~ 100 kDa and a prominent smaller band at ~ 25 kDa representing the N-terminal fragment of the furin-processed subunit due to cleavage at two consensus furin cleavage sites RSRR$\downarrow$^178^ and RRAR$\downarrow$^201^ (Fig. 2b). The observed band patterns are larger than the expected peptide sizes, which is likely due to glycosylation [26] [37]. The γ*-subunit showed two bands, one at ~ 100 kDa, representing a full-length fragment, and a second at ~ 20 kDa, likely a product of furin-mediated cleavage at RKRR$\downarrow$^140^ of the γ-subunit. For the δ*-subunit, on the other hand, only one band at ~ 70 kDa, corresponding to the full-length peptide, was detected. This subunit lacks a minimal furin cleavage consensus site [11]. These observations were confirmed by targeting the C-terminal V5 tags. The anti-V5 immunoblots revealed two bands for the α*-subunit, full-length peptide at ~ 100 kDa and a broader signal at ~ 65 kDa (representing subunit cleavage). The γ*-subunit showed the full-length peptide at ~ 100 kDa and a second band at ~ 80 kDa, while the δ*-subunit showed only the full-length band at ~ 70 kDa. These data indicate that the guinea pig α-ENaC and γ-ENaC subunits are processed by furin, whereas the δ-subunit is not.

The addition of the tags to these subunits did not alter ENaC activity except when the γ*-subunit was expressed in combination with the α- and β-subunits, where the ΔI_Ami_ of oocytes expressing this construct was slightly lower in comparison to those expressing untagged ENaCs (Fig. 2c). However, the presence of the tags did not alter the response to chymotrypsin (Fig. 2d). Chymotrypsin treatment significantly activated all αβγ-ENaC constructs, and this effect was independent of subunit tagging. The δβγ-ENaCs, on the other hand, remained unresponsive to chymotrypsin, regardless of tagging. Since the response to proteases was unaltered and the tags could be detected on the immunoblots, these tagged ENaC constructs were used for further evaluation of subunit processing by proteases.

### The γ-subunit of Guinea pig δβγ-ENaC is cleaved by extracellular chymotrypsin

**Fig. 3:**
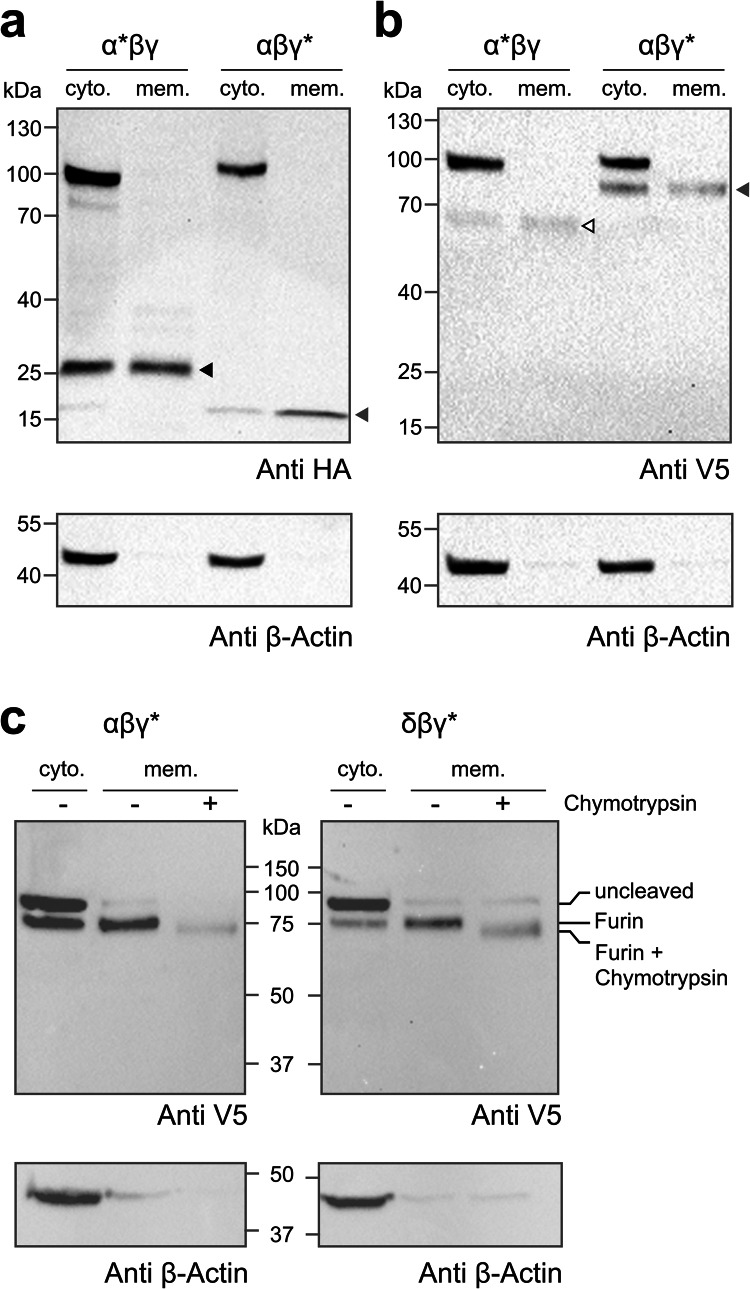
The γ-subunit of guinea pig δβγ-ENaC is cleaved by extracellular chymotrypsin. (**a,b**) Immunoblots following separation of membrane (mem.) from cytosolic (cyto.) fractions of oocytes expressing α*βγ-ENaC, or αβγ*-ENaCs. Blots were probed with either an anti-HA **(a)** or an anti-V5 (**b**) antibody. Both the full-length and furin-cleaved fragments of the α*- and γ*-subunits could be detected in the cytosolic fractions, only furin-cleavage products were present in membrane fractions. The bottom blots were obtained after reprobing the same membranes with an anti-β-actin antibody to confirm proper separation of membrane from cytosolic fractions. Filled and unfilled black arrowheads indicate fragments due to proximal and distal furin-mediated cleavage in the α*-subunit as shown by the same arrowheads in Fig. 2a. Filled grey arrowheads indicate fragments due to furin-mediated cleavage in the γ*-subunit as shown by the same arrowhead in Fig. 2a. The blots represent one of *n* = 3 independent experimental repeats. Original uncropped versions of the gels and blot images shown in these panels are provided in Supplementary Figures 4 and 5. (**c**) To evaluate the ability of extracellular chymotrypsin to cleave the γ-subunit, oocytes were injected with αβγ*-ENaC or δβγ*-ENaC cRNAs. Half the number of oocytes injected per construct were treated with chymotrypsin (2 µg/ml) before biotinylation and immunoblotting. Upon chymotrypsin treatment, there was a detectable decrease in the molecular mass of the processed γ*-subunit in both ENaC isoforms. Reprobing with anti-β-actin antibody was used to confirm separation of membrane from cytosolic fractions. The blots represent one of *n* = 3 independent experimental repeats. Original uncropped versions of the gels and blot images shown in this panel are provided in Supplementary Figure 6.

Furin-mediated cleavage of ENaC subunits occurs in the trans-Golgi network. As such, the majority of ENaCs found at the cell surface are expected to have undergone furin cleavage already. To evaluate the cleavage status of membrane-located ENaCs, surface biotinylation experiments were performed on  *Xenopus* oocytes expressing α*βγ-ENaC, αβγ*-ENaC, or δβγ*-ENaC. The presence of β-actin was used to assess the efficiency of the separation of membrane fractions from cytosolic fractions (Fig. 3a). Only furin-processed ENaCs were detectable at the cell membrane. Anti-HA immunoblots targeting α*-subunits revealed a band at ~ 100 kDa and a smaller band at ~ 25 kDa in the cytosolic fractions. The membrane fraction showed only a single band at ~ 25 kDa. In the γ*-subunits, a band at ~ 100 kDa and a smaller ~ 20 kDa band were detected in the cytosolic fractions. Only the ~ 20 kDa band could be detected in the membrane fractions. Anti-V5 blots confirmed these observations (Fig. 3b). The cytosolic α*-subunits showed bands at ~ 100 kDa and ~ 65 kDa but only a single ~ 65 kDa band could be seen in the membrane fractions. The γ*-subunits had bands at ~ 100 kDa and ~ 80 kDa in the cytosolic fractions but only the ~ 80 kDa band could be detected in the membrane fractions.

We have previously shown in amphibian ENaC orthologues that extracellular chymotrypsin was unable to cleave the γ-ENaC subunit in the presence of the δ-subunit [37]. To evaluate whether this phenomenon also applies to mammalian (guinea pig) ENaCs, half the population of oocytes expressing αβγ*-ENaC, and δβγ*-ENaC were incubated for 5 min at room temperature in a NMDG-ORS containing 2 µg/ml of chymotrypsin, prior to surface biotinylation to separate the membrane from cytosolic fractions and immunoblotting using an anti-V5 antibody. At the cell membrane, αβγ*-ENaC showed one band for the γ*-subunit at ~ 80 kDa (Fig. 3c). Exposure to chymotrypsin resulted in a reduction in the band size to ~ 75 kDa, consistent with chymotrypsin-mediated cleavage. In guinea pig δβγ*-ENaC, a similar size reduction in γ*-subunit from ~ 80 kDa to ~ 75 kDa after treatment with chymotrypsin was observed (Fig. 3c). These data indicate that extracellular proteases can cleave the γ-ENaC subunit in both αβγ- and δβγ-ENaC assemblies. While this cleavage event activates αβγ-ENaC, there is no effect on δβγ-ENaC activity, as shown in Fig. 1.

### The dominant role of γ-subunit cleavage on ENaC activation is absent in guinea pig δβγ-ENaC

**Fig. 4:**
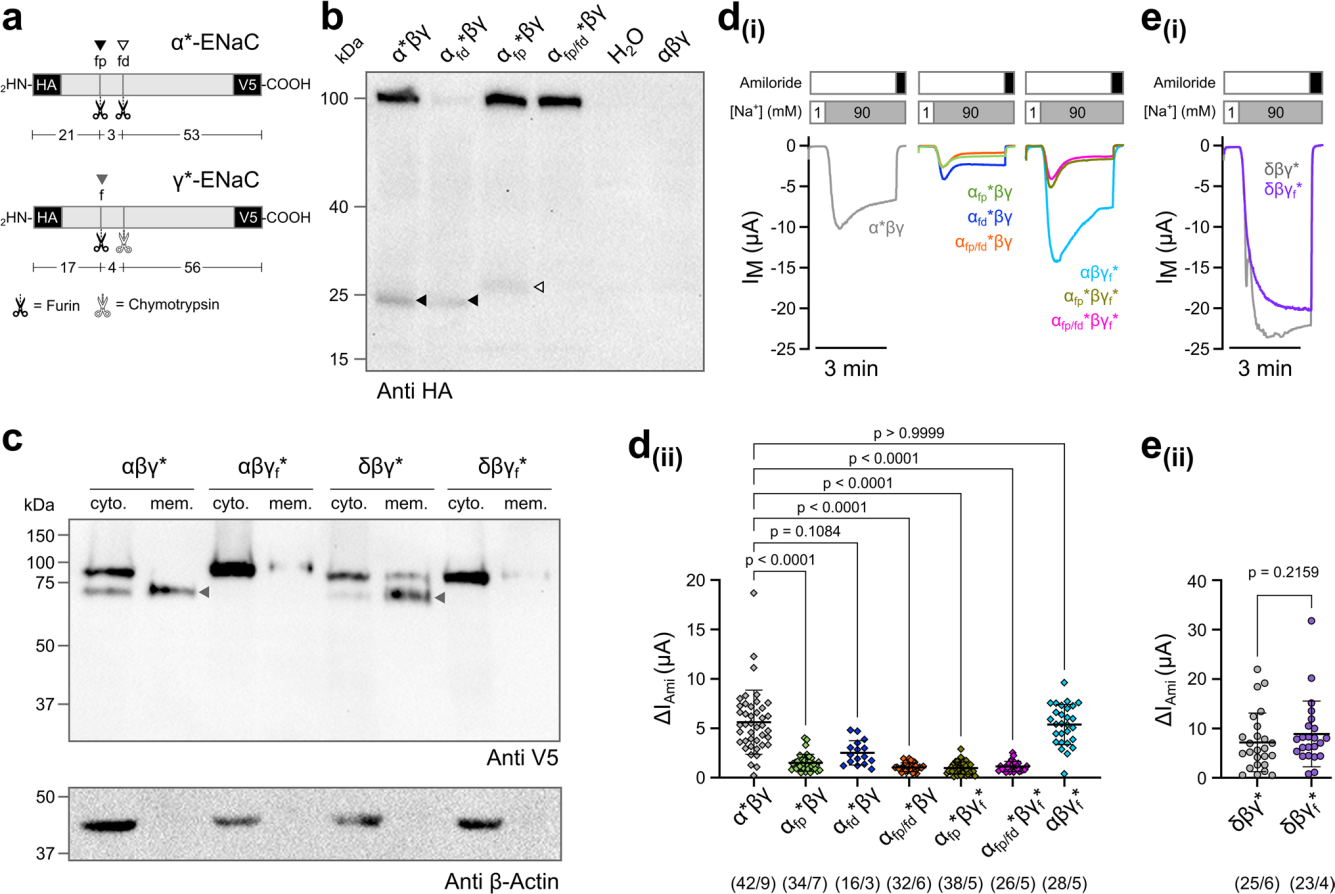
Removal of furin consensus sites in the guinea pig α- and γ-subunits prevents furin-mediated subunit processing. (**a**) Schematic depiction of epitope-tagged guinea pig ENaC subunits (grey bars) containing N-terminal HA- and C-terminal V5-tags (black bars). Numbers indicate the molecular mass (kDa) of the peptide fragments, while the scissors indicate where proteases cleave the subunits. Asterisks indicate the presence of both HA- and V5 tags in a subunit. ENaCs carrying mutations in the proximal and distal furin cleavage sites of the α-subunit are indicated with ‘fp’ and ‘fd’, respectively. ENaCs carrying the mutation of the γ-subunit’s furin cleavage site are indicated with an ‘f’. (**b**) Immunoblot of whole-cell lysates from oocytes expressing α*βγ-ENaC probed with an anti-HA antibody. The putative furin cleavage sites were sequentially removed, first the distal (α_fd_*βγ) and proximal furin cleavage site (α_fp_*βγ) and then both sites (α_fp/fd_*βγ). In α_fp_*βγ-ENaC and α_fp/fd_*βγ the band at ~ 25 kDa (black arrowhead) disappeared, confirming that cleavage at the proximal site had been impaired. The fragment resulting from cleavage at the distal furin cleavage site in α_fp_*βγ is labelled with an unfilled arrowhead. Oocytes expressing untagged αβγ-ENaC and water-injected oocytes (H_2_O) served as controls. The blot represents one of *n* = 3 independent experimental repeats (for tagged ENaC proteins). Original uncropped versions of the gels and blot images shown in this panel are provided in Supplementary Figure 7. (**c**) Immunoblots on membrane (mem.) and cytosolic (cyto.) factions derived from oocytes expressing αβγ*-, αβγ_f_*-, δβγ*- and δβγ_f_*-ENaC probed with an anti-V5 antibody. The product (grey arrowhead) resulting from furin cleavage of the γ-subunit disappeared in both αβγ_f_*- and δβγ_f_*-ENaC assemblies. The bottom blot shows the results after reprobing the same membrane with an anti-β-actin antibody to confirm proper separation of membrane from cytosolic fractions. The blot represents one of *n* = 3 independent experimental repeats. Original uncropped versions of the gels and blot images shown in this panel are provided in Supplementary Figure 8. (**d**_**(i)**_) Representative transmembrane current (I_M_) traces derived from TEVC recordings of oocytes expressing αβγ-ENaCs. Furin cleavage sites were removed from the α*- (α_fp_*, α_fd_*, α_fp/fd_*) and γ*-subunits (γ_f_*). ENaC-mediated current fractions were determined by switching extracellular solutions containing low (1 mM) to high (90 mM) [Na^+^] and application of the ENaC-inhibitor amiloride (100 µM, black bars). V_M_ = −60 mV. (**d**_**(ii)**_) Comparison of the amiloride-sensitive current fractions (ΔI_Ami_) derived from experiments shown in panel (d_(i)_). Statistical analyses were done with Kruskal–Wallis test followed by Dunn’s multiple-comparison test. (**e**_**(i)**_) Representative I_M_ traces derived from TEVC recordings of oocytes expressing δβγ*-ENaCs with and without disruption of the furin cleavage site in the γ*-subunit (γ_f_*). (**e**_**(ii)**_) Comparison of ΔI_Ami_ derived from experiments shown in panel (e_(i)_). Statistical analysis was done with Mann–Whitney U-test. Numbers in parentheses indicate oocytes/oocyte donors

In several ENaC orthologs, it has been shown that furin-mediated cleavage of αβγ-ENaC is directly coupled to ENaC activity. Removal of the inhibitory tract from the α-subunit by furin increases channel P_o_, while cleavage of the γ-subunit proximal to its inhibitory peptide is pivotal for further activation by extracellular proteases and yields channels with a very high P_o_ [9, 16, 30, 37]. Consistently, when the proximal furin consensus site in N-terminally HA- and C-terminally V5-tagged guinea pig α-ENaC was mutated alone (α_175QQQQ178_*_,_ α_fp_* Fig. 4a) and expressed together with untagged β- and γ-ENaC, the ~ 25 kDa fragment shifted to a larger molecular weight (Fig. 4b). When this site was mutated together with the distal furin consensus site (α_175QQQQ178,/198QQQQ201_*, α_fp/fd_*, Fig. 4a), the cleavage fragment at ~ 25 kDa largely disappeared (Fig. 4b). Mutation of the N-terminally HA- and C-terminally V5-tagged guinea pig γ-subunit’s furin cleavage site (γ_137QQQQ140_*, γ_f_*, Fig. 4a) also abolished cleavage and yielded only full-length γ-subunit fragments (Fig. 4c). While the ΔI_Ami_ was reduced in oocytes expressing guinea pig ENaC containing furin-cleavage site disruptions in the α-subunit (α_fp_*, α_fd_*, α_fp/fd_*), there was no altered ΔI_Ami_ when only the γ-subunit containing a disrupted furin-cleavage site (γ_f_*) was present (Fig. 4d). Interestingly, in oocytes expressing ENaC containing a disrupted distal furin cleavage site in the α-subunit (α_fd_*), the ΔI_Ami_ were larger than in those expressing ENaC with the disrupted proximal cleavage site (α_fp_*, Fig. 4d). Unlike human ENaC, guinea pig α-ENaC contains an additional arginine-rich sequence (α*^214^RVDRRDWR^221^, Supplementary Figure 7b) downstream of the distal furin cleavage site that harbours two potential and partially overlapping furin cleavage sites (RVDRR and RRDWR). Computational prediction of furin-specific cleavage using ProP 1.0 (accessed 08.03.2026) [10] reveals cleavage scores of 0.254/0.343 (RVDRR) and 0.219 (RRDWR) for this region. Although these are lower than the scores for the canonical proximal (RSRR$\downarrow$^178^, 0.720) and distal (RRAR$\downarrow$^201^, 0.522) furin cleavage sites, this sequence could potentially be cleaved by furin in an overexpression system. This could occasionally release the α-subunit’s inhibitory peptide and thereby increase ΔI_Ami_. In guinea pig δβγ-ENaCs, disruption of the furin cleavage site from the γ-subunit (γ_f_*) had also no impact on the ΔI_Ami_ of guinea pig δβγ*-ENaC expressing oocytes (Fig. 4e).

**Fig. 5:**
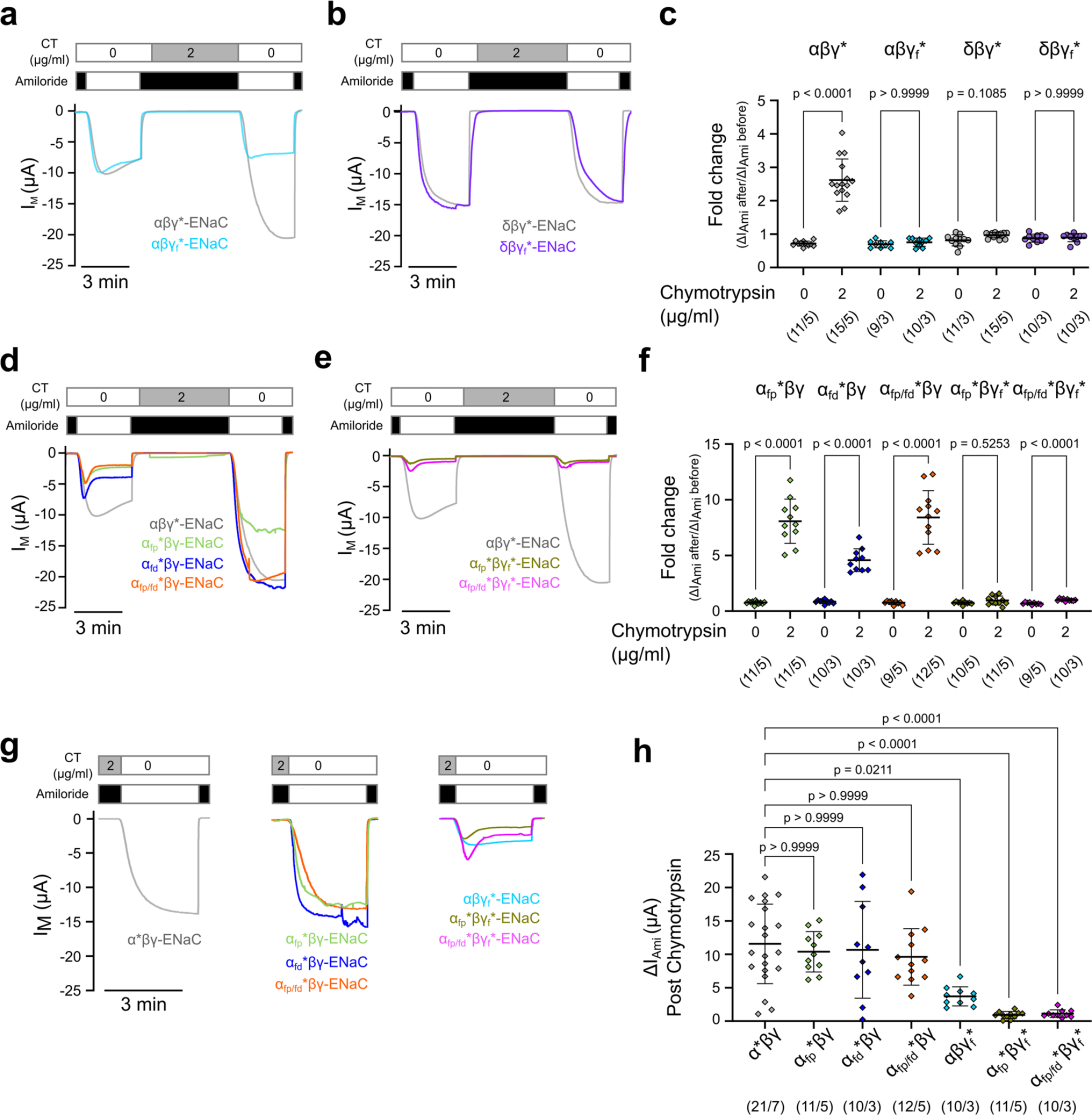
The dominant role of γ-subunit cleavage on ENaC activation is absent in guinea pig δβγ-ENaC. (**a, b**) Representative I_M_ traces from TEVC recordings of oocytes expressing αβγ*-ENaC (**a**) and δβγ*-ENaC (**b**). Overlayed are the traces from oocytes expressing αβγ_f_*-ENaC (light blue, a) and δβγ_f_*-ENaC (magenta, b). The oocytes were incubated in chymotrypsin (CT) together with amiloride (100 µM, black bars) for 5 min, and the impact of the protease on ENaC activity was evaluated by comparing the amiloride-sensitive current fractions (ΔI_Ami_) before and after the application of chymotrypsin. (**c**) Comparison of the fold change in ΔI_Ami_ between the chymotrypsin-treated experimental groups as shown in panels (a/b) and control groups without application of chymotrypsin. Kruskal–Wallis test and Dunn’s multiple-comparison tests were used for statistical analysis. (**d-f**) Representative I_M_ traces (d/e) and statistical analysis of fold change in ENaC activity in response to chymotrypsin (f) derived from TEVC experiments similar to those shown in panels (a/b) using oocytes expressing αβγ-ENaC with or without mutated furin cleavage sites in the α- and γ-subunits. Note that for comparison, the trace for αβγ*-ENaC shown in panels (d/e) is identical to the one shown in panel (a). Kruskal–Wallis test and Dunn’s multiple-comparison test were used for statistical analysis. (**g**) Representative I_M_ traces derived from TEVC experiments similar to those shown in panels (a, d and e), comparing amiloride-sensitive current fractions (ΔI_Ami_) after treatment with chymotrypsin (CT). (**h**) Statistical analysis of ΔI_Ami_ derived from experiments shown in panel (**g**). Ordinary one-way ANOVA and Sidak’s multiple-comparison test were used for statistical analysis. Data points in the scatter plots are derived from *n* individual experiments. Lines and error bars indicate the mean and standard deviation of the mean. Absolute values of ΔI_Ami_ before and after protease (or mock experiments) are provided in Supplementary Figure 9. Numbers in parentheses indicate oocytes/oocyte donors

Removal of the γ-subunit’s inhibitory tract plays a critical role in the activation of αβγ-ENaC by extracellular proteases. Consistently, disruption of the furin cleavage site in the γ-subunit (γ_f_*-ENaC), which prevents removal of the inhibitory tract by extracellular proteases, abolished the chymotrypsin-mediated activation of ΔI_Ami_ in guinea pig αβγ*-ENaC, but had no effect on δβγ*-ENaC assemblies (Fig. 5a-c).

In αβγ-ENaC, cleavage of the γ-subunit by extracellular proteases (and removal of the γ-subunit’s inhibitory tract) can increase P_o_ to maximum levels despite the presence of the α-subunit’s inhibitory tract [5]. Consistent with this concept, chymotrypsin increased ΔI_Ami_ of oocytes expressing α_fp/fd_*βγ-ENaC to the same levels as those expressing α*βγ-ENaC but had no effect on ENaC subunit combinations containing the γ_f_*-subunit (Fig. 5d-h). When only the distal cleavage site in the α-subunit (α_fd_*) was disrupted, the fold-activation in response to chymotrypsin was smaller compared to experiments where only the proximal (α_fp_*) or the proximal and distal (α_fp/fd_*) cleavage sites were disrupted (Fig. 5f**)**. This is again consistent with a potential occasional cleavage at the arginine-rich sequence downstream of the distal furin cleavage site and release of the α-subunit’s inhibitory tract. Overall, these data indicate that the presence of the α-subunit’s inhibitory tract determines baseline activity (i.e. P_o_) of guinea pig αβγ-ENaC, while removal of the γ-subunit’s inhibitory tract controls maximum channel activation. In guinea pig δβγ-ENaCs, cleavage and/or removal of the γ-subunit’s inhibitory tract did not affect channel activity.

### The enlarged ‘knuckle’ domain of guinea pig δβγ-ENaC does not affect channel activity and response to extracellular protease

**Fig. 6:**
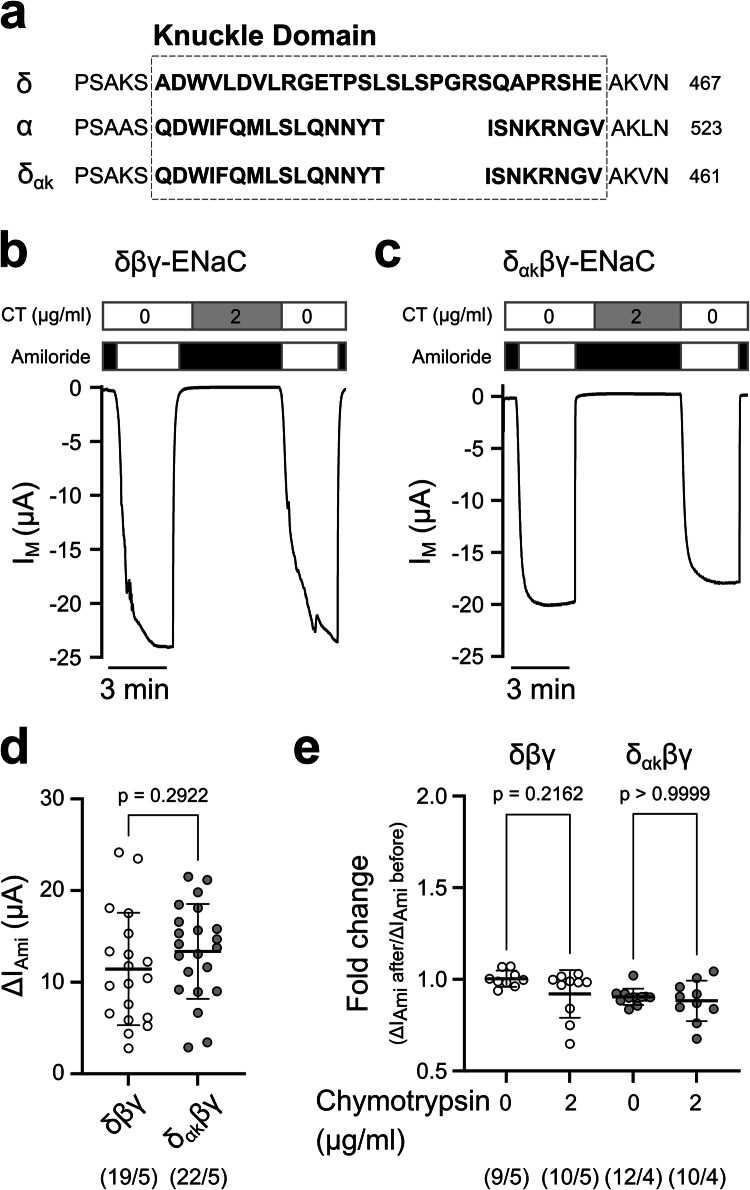
The extended knuckle domain in the guinea pig δ-ENaC subunit is not sufficient to explain protease-insensitivity in δβγ-ENaC. **(a)** Alignment of the knuckle domains in the guinea pig δ- and α-subunits and a δ-subunit construct containing the knuckle sequence of the α-subunit (δ_αk_). (**b,c**) Representative transmembrane current (I_M_) traces obtained from TEVC recordings of oocytes expressing guinea pig δβγ-ENaC (**b**) and δ_αk_βγ-ENaC (**c**) with V_M_ = −60 mV. Amiloride (100 µM, black bars) was used to determine ENaC-mediated amiloride-sensitive current fractions (ΔI_Ami_). Oocytes were incubated for 5 min in chymotrypsin (2 µg/ml, CT) in the presence of amiloride, and the impact of the protease on ΔI_Ami_ after the application of chymotrypsin was recorded. (**d**) Comparison of the ΔI_Ami_ between oocytes expressing guinea pig δβγ-ENaC and δ_αk_βγ-ENaC (without protease treatment). Values were obtained from the first application of amiloride in experiments as shown in panels (b/c). Statistical analysis was performed with unpaired t-test with Welch’s correction. (**e**) Comparison of the fold change in ΔI_Ami_ between the chymotrypsin-treated experimental groups as shown in panels (b/c) and control groups without application of chymotrypsin. Statistical analyses were performed with Kruskal–Wallis test followed by Dunn’s multiple-comparison test. Absolute values of ΔI_Ami_ before and after protease (or mock experiments) are provided in Supplementary Figure 10. Numbers in parentheses indicate oocytes/oocyte donors

A characteristic structural feature of the guinea pig δ-ENaC subunit is an enlarged ‘knuckle’ domain, which is the result of the fusion of exons 11 and 12 and incorporation of intron DNA into the δ-ENaC coding sequence in the rodent suborder Hystricomorpha [11]. Recent cryo-EM analyses on human ENaC assemblies suggest that the δ-subunit knuckle domain can influence the GRIP domain of the adjacent γ-subunit [15]. The additional amino acid residues introduced by exon fusion in the guinea pig δ-subunit could potentially affect the conformational flexibility of the neighbouring γ-subunit’s GRIP domain. Such steric interference could thereby contribute to the lack of proteolytic activation of guinea pig δβγ-ENaC. We therefore replaced the ‘knuckle’ domain of the guinea pig δ-ENaC subunit by that of the α-subunit (δ_αk_) (Fig. 6a). However, this replacement was not sufficient to alter ΔI_Ami_ of oocytes expressing δ_αk_βγ-ENaC (13.36 ± 5.18 μA, *n* = 22) compared with those expressing δβγ-ENaC (11.45 ± 6.13 μA, *n* = 19) (Fig. 6b-d). Furthermore, extracellular chymotrypsin had no effect on ΔI_Ami_ of oocytes expressing δ_αk_βγ-ENaC (Fig. 6b,c,e). Oocytes expressing δ_αk_βγ-ENaC showed a fold change of 0.90 ± 0.04 (*n* = 12) under control conditions, which was not statistically different from the fold change of 0.88 ± 0.11 (*n* = 10; p > 0.9999) following treatment with chymotrypsin (Fig. 6e). This observation matched what was seen for the δβγ-ENaC expressing oocytes where a fold change of 1.00 ± 0.04 (*n* = 9) under control conditions was not statistically significantly different from the 0.92 ± 0.13 (*n* = 10, *p* = 0.2162) fold change under chymotrypsin treatment conditions (Fig. 6e). These results suggest that the enlarged ‘knuckle’ domain is not sufficient to explain the lack of proteolytic activation of guinea pig δβγ-ENaC.

### Guinea pig δβγ-ENaC has an intrinsically high open probability

**Fig. 7:**
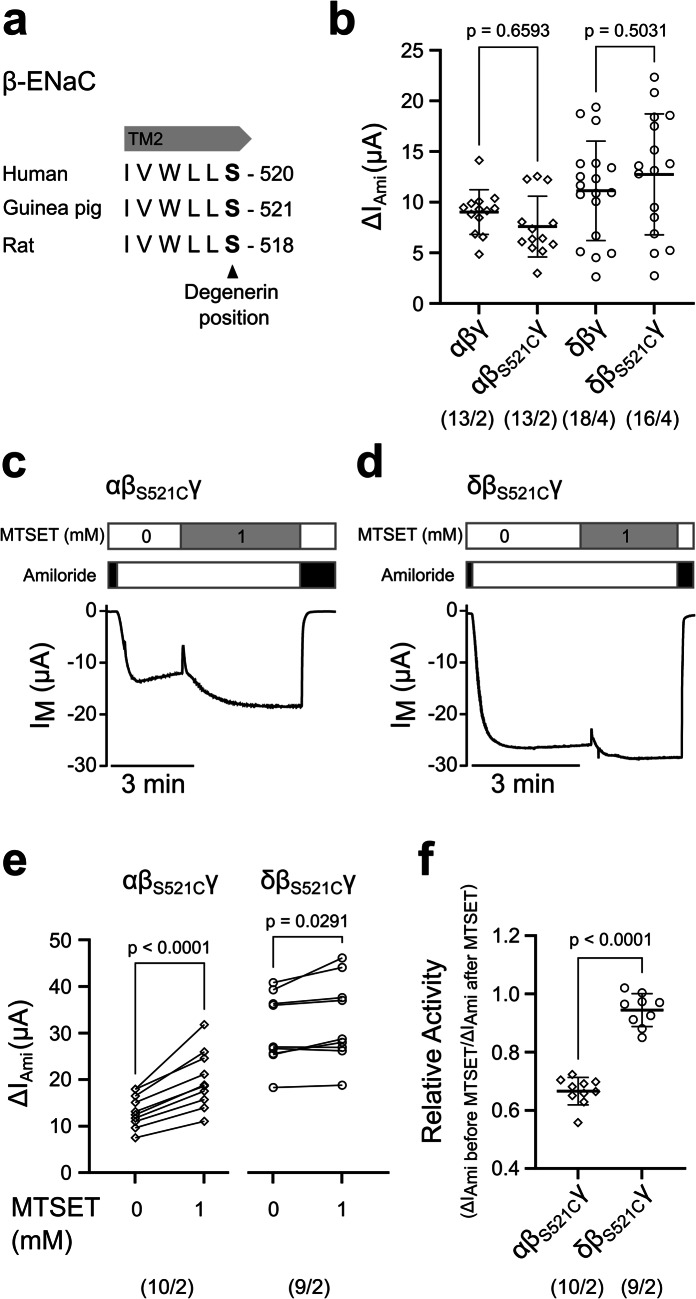
Guinea pig δβ_S521C_γ-ENaC has an intrinsically high activity. (**a**) Indication of the conserved degenerin-site at the beginning of the second transmembrane domain (TM2) of human, guinea pig and rat β-ENaC subunit. The serine at position 521 in guinea pig β-ENaC was substituted by cysteine. (**b**) Amiloride-sensitive transmembrane currents (ΔI_Ami_) derived from TEVC recordings of oocytes expressing αβγ-ENaC and δβγ-ENaC with or without the β_S521C_ substitution. V_M_ = −60 mV. Ordinary one-way ANOVA and Sidak’s multiple-comparison tests were used for statistical analysis. Data points in the scatter plots are derived from *n* individual experiments. Lines and error bars indicate the mean and standard deviation of the mean. (**c,d**) Representative transmembrane current (I_M_) traces derived from TEVC recordings of oocytes expressing αβ_S521C_γ- or δβ_S521C_γ-ENaC with V_M_ = −60 mV. Amiloride (100 µM, black bars) was used to determine ENaC-mediated amiloride-sensitive current fractions (ΔI_Ami_) before and after application of MTSET (1 mM, grey bars). (**e**) Statistical analysis of ΔI_Ami_ determined from experiments as shown in panels (c/d) before (0) and after (1) application of MTSET. Statistical analyses were performed with Student’s paired t-test. (**f**) Relative activity of αβ_S521C_γ- or δβ_S521C_γ-ENaC determined from the ratio ΔI_Ami_ before MTSET/ΔI_Ami_ after MTSET. Statistical analysis was performed with Mann–Whitney U-test. Data points in the scatter plots are derived from *n* individual experiments. Lines and error bars indicate the mean and standard deviation of the mean. Numbers in parentheses indicate oocytes/oocyte donors

The lack of protease-mediated activation of guinea pig δβγ-ENaC and the lack of channel activation after removal of the γ-subunit’s inhibitory tract might suggest that the presence of the δ-subunit locks ENaC in an open state that cannot be further enhanced by proteolytic cleavage of the γ-subunit. To evaluate the overall open probability (P_o_) in guinea pig ENaC isoforms, a β_S521C_ mutation was introduced into the conserved degenerin site of the β-subunit (Fig. 7a) and expressed in combination with α- and γ-subunits, or with δ- and γ-subunits. This procedure has been previously employed to estimate the P_o_ of human [13, 31], rat [20] and amphibian [37] ENaCs. The introduction of the β_S521C_ mutation did not affect ENaC activity since the ΔI_Ami_ of oocytes expressing ENaC constructs with the β_S521C_ mutation was not different from those expressing wild-type ENaCs (Fig. 7b). When ENaC containing the introduced cysteine is treated with 1 mM [2-(trimethylammonium)ethyl] methanethiosulfonate (MTSET; Biotium, Fremont, CA, USA), the sulfhydryl reagent covalently modifies the cysteine and destabilises the closed state of the channel [8]. This locks the channels at a P_o_ of nearly 1.0. In this experimental setup, a comparison of the ΔI_Ami_ before and after MTSET application allows estimation of the relative activity of all ENaCs expressed in the oocyte.

The application of MTSET increased transmembrane currents (I_M_) in both αβ_S521C_γ- and δβ_S521C_γ-ENaC expressing oocytes, but the increase was much smaller in those expressing δβ_S521C_γ- than αβ_S521C_γ-ENaC (Fig. 7c-e). Comparison of ΔI_Ami_ before to ΔI_Ami_ after MTSET application revealed a significantly higher relative activity for the δβ_S521C_γ-ENaC channels (0.94 ± 0.06, *n* = 9) as compared to the αβ_S521C_γ-ENaC channels (0.67 ± 0.05, *n* = 10, *p* < 0.0001) (Fig. 7f). Assuming that MTSET increased the P_o_ of both ENaC isoforms to 1.0 without changing the unitary conductance, this suggests that guinea pig δβ_S521C_γ-ENaCs are nearly fully open when expressed in *Xenopus* oocytes.

**Fig. 8:**
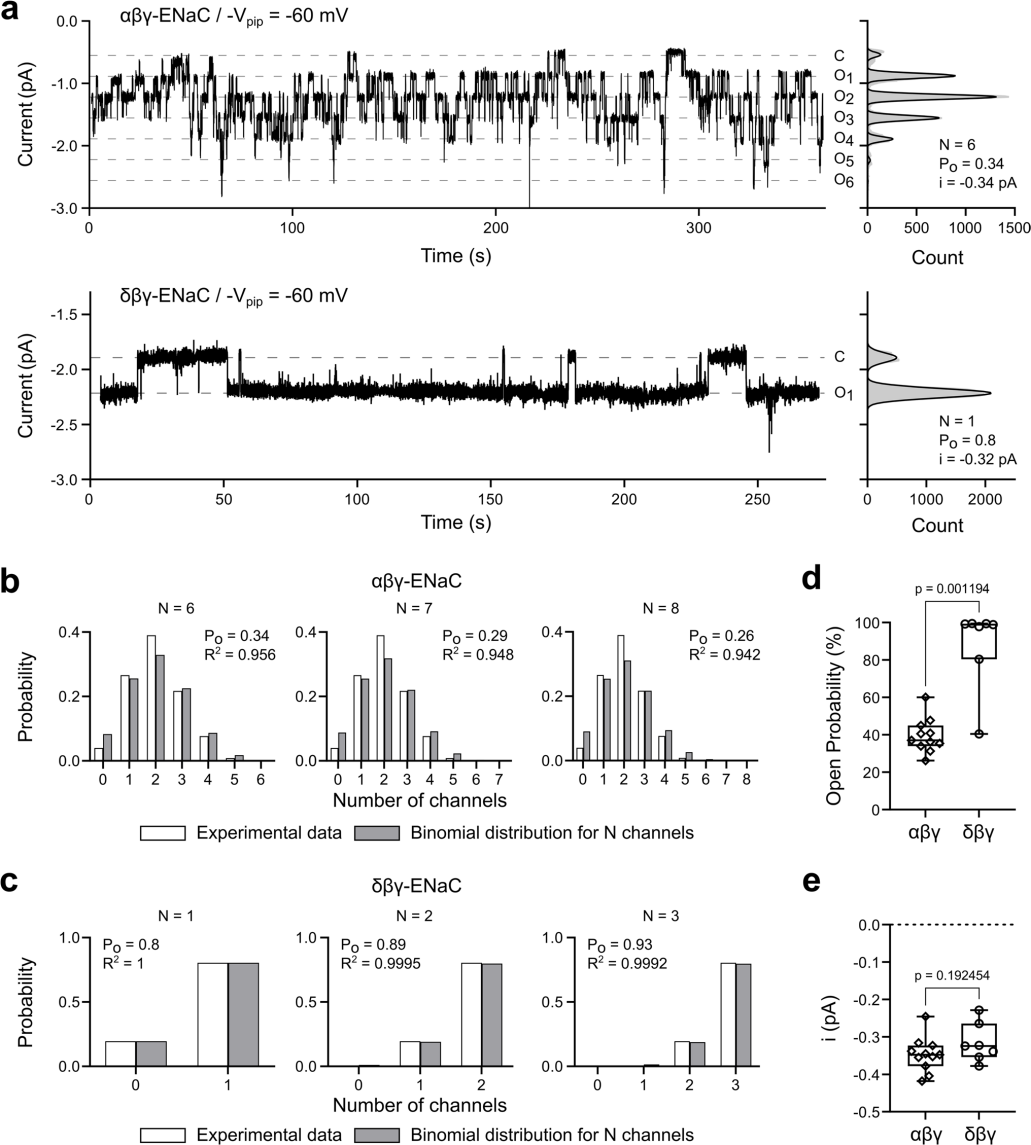
Guinea pig δβγ-ENaC shows a higher open probability than αβγ-ENaC in on-cell patch-clamp recordings. (**a**) Representative current traces (left) from oocytes expressing αβγ- (top) and δβγ-ENaC (bottom) and corresponding amplitude histograms (right). The current traces were recorded using the patch-clamp technique in the on-cell configuration at -V_pip_ = −60 mV. Dashed grey lines mark the different current levels resulting from the independent opening and closing of the ENaC channels in the membrane patch. The baseline level (no channel open) is indicated with C. The current levels resulting from the simultaneous opening of *k* channels are indicated with O_k_. The amplitude histograms were fitted with a sum of Gaussian functions (solid black line) to determine the current levels and occupation probabilities. The number of channels (N), the open probability (P_o_), and the open-channel amplitude (i) are shown in the graphs. (**b**) Occupation probabilities of the different current levels of the αβγ-ENaC recording shown in (a) (white bars) were fitted with binomial distributions (grey bars) for *N* = 6 to *N* = 8 channels, assuming that all channels are in the closed state at the lowest observed current level (level C in (a)). The calculated open probabilities (P_o_) and the R^2^ values of the best fits are given in the corresponding graphs. (**c**) Occupation probabilities of the two observed current levels of the δβγ-ENaC recording shown in (a) (white bars) were fitted with binomial distributions (grey bars) for *N* = 1 to *N* = 3 channels, assuming that all channels are in the open state at the highest observed current level (i.e. the current level with the largest amplitude). The calculated P_o_ and the R^2^ values of the best fits are given in the corresponding graphs. (**d**) Open probabilities calculated from recordings as shown in panel (a). Statistical comparison was performed using a Mann–Whitney U-test. (**e**) Single-channel current amplitudes calculated from recordings as in (a). Statistical comparison was performed using Student’s unpaired, two-tailed t-test. Data points in the box plots in (d) and (e) are derived from *n* = 11 (8 oocytes, 6 donors) and* n* = 7 (5 oocytes, 3 donors) individual on-cell patch-clamp recordings for αβγ- and δβγ-ENaC, respectively. Boxes of the box plots represent the 25th and 75th percentile; the median (50th percentile) is shown as horizontal line. The bars indicate the minimal and maximal values

To confirm these observations on native guinea pig ENaCs, on-cell patch-clamp experiments were performed on devitellinised oocytes expressing αβγ-ENaC or δβγ-ENaC to assess single-channel activity. Figure 8a shows representative current traces and corresponding amplitude histograms from αβγ- and δβγ-ENaC-expressing oocytes recorded at a pipette holding potential -V_pip_ = −60 mV in the on-cell configuration. The current trace and amplitude histogram of the αβγ-ENaC-expressing oocyte (Fig. 8a, top) shows seven equidistant current levels between which the current fluctuates, with the upper level representing the baseline (= all channels closed, C) and the six lower current levels (O_1_ to O_6_) representing the simultaneous opening of 1 to 6 ENaCs. The existence of six open channel levels indicates the presence of at least six αβγ-ENaCs. To determine the occupation probabilities of the seven current levels, the amplitude histogram was fitted with the sum of seven Gaussian functions (Eq. 1). Assuming that the observed current fluctuations are caused by the presence of *N* = 6 identically behaving αβγ-ENaCs, fitting with a binomial distribution (Eq. 4, Fig. 8b) yields an open probability of 34%. Since it cannot be ruled out that the observed current fluctuations were caused by more than six channels, this fit was repeated for *N* = 7 and *N* = 8 channels (Fig. 8b). As can be seen from the coefficients of determination (R^2^), binomial distributions for *N* = 7 and *N* = 8 do not describe the experimental data any better. However, it is important to note that the estimated open probability decreases as the assumed number of channels increases. Hence, if it is assumed that the number of ion channels in a patch corresponds to the number of observed open channel levels, the open probability is likely to be overestimated. The mean open probability calculated in this way from *n* = 11 independent recordings is P_o_ = 39.5 ± 8.7% (Fig. 8d), with an average of *N* = 4.6 ± 1.4 channels in each patch.

The representative δβγ-ENaC measurement shown in Fig. 8a shows only two current levels. Assuming that only one channel was present in the patch, the occupation probability of the lower current level corresponds to the open probability, which could be calculated as P_o_ = 80%. Interestingly, all other δβγ-ENaC recordings also showed only two current levels. Assuming that there was indeed only one channel in each membrane patch in all these recordings, the mean P_o_ is 87.9 ± 20.4% (*n* = 7) (Fig. 8d). However, since the amiloride-sensitive whole-cell currents of δβγ-ENaC-expressing oocytes were on average 2.5 times larger than the currents of αβγ-ENaC-expressing oocytes (Fig. 2c), it seems rather unlikely that on average less channels are found in the measured membrane patches of δβγ-ENaC-expressing oocytes. It is therefore likely that the open channel level identified as O_1_ in the on-cell recordings represents the current from two or more simultaneously open δβγ-ENaCs. The open probability of δβγ-ENaC is therefore very likely to be even higher. To demonstrate this, we fitted the occupation probabilities calculated from the δβγ-ENaC recording in Fig. 8a with binomial distributions for *N* = 1, 2, and 3. The fitting results in Fig. 8c show that P_o_ increases with the number of assumed channels (P_o_ = 0.80, 0.89 and 0.93 for *N* = 1, 2 and 3, respectively) with little change in the quality of the fit (R^2^ = 1, 0.9995 and 0.9992 for *N* = 1, 2 and 3, respectively).

Consistent with our previous report [11], the analysis of the on-cell patch-clamp recordings also revealed that both αβγ- and δβγ-ENaC have almost identical single-channel amplitudes with i = −0.35 ± 0.04 pA and i = −0.32 ± 0.05 pA, respectively (Fig. 8e). In summary, guinea pig δβγ-ENaCs are locked in an almost completely open state, thereby explaining the lack of chymotrypsin-induced channel activation in comparison to the αβγ-ENaC subunit assembly.

## Discussion

Despite cloning of δ-ENaC from human kidney in 1995 [33], limited progress has been made in understanding the physiological and potentially pathophysiological role of ENaCs containing this subunit. A major reason is the absence of a suitable mammalian animal model, as the *SCNN1D* gene coding for δ-ENaC is a pseudogene in traditional model organisms such as rats and mice [11]. Recently, a *SCNN1D* knock-in transgenic mouse was generated; however, while this study demonstrated a potential role for δ-ENaC in lung transepithelial sodium and liquid transport, this animal model does not reflect physiological conditions [17]. Furthermore, there are currently no specific modulators available which would allow precise functional experimental discrimination of different ENaC subunit assemblies in situ and in vivo. Therefore, it is important to determine which mammalian species contain *SCNN1D* genes and functionally characterise the corresponding δ-ENaC subunits as a prerequisite for establishing appropriate model systems that enable future in situ and in vivo studies.

We have previously demonstrated that guinea pigs (*Cavia porcellus*) contain a *SCNN1D* gene that encodes the δ-ENaC subunit capable of forming functional δβγ-ENaCs when co-expressed with the β- and γ-subunits [11]. Building upon this work, the present study extends the functional characterization of guinea pig δβγ-ENaC, with a particular focus on its regulation by proteases. We heterologously expressed guinea pig αβγ-ENaC or δβγ-ENaC in *Xenopus* oocytes and assessed ENaC activity by TEVC and patch-clamp electrophysiology. The application of proteases (chymotrypsin and trypsin) robustly activated αβγ-ENaC while there was no such effect on δβγ-ENaC. These observations are consistent with previous studies which demonstrated that replacing the α-subunit with the δ-subunit either reduced sensitivity or rendered ENaC completely insensitive to extracellular proteases: Human δβγ-ENaC is activated by extracellular chymotrypsin, but to a much lower extent to that observed in αβγ-ENaC [11, 13]. Similar to our observations on guinea pig δβγ-ENaC, amphibian δβγ-ENaC from *Xenopus laevis* is completely insensitive to extracellular chymotrypsin [37]. These data suggest that proteolytic ENaC activation is highly conserved in αβγ-ENaC, but largely reduced or absent in δβγ-ENaC assemblies.

Immunoblot analyses of epitope-tagged guinea pig ENaC subunits suggest two furin-mediated cleavage events in the α-subunit which are consistent with the presence of furin consensus sites within the subunit’s GRIP domain [11], while the δ-ENaC subunit showed only full-length peptides consistent with the lack of furin consensus sites [11, 35]. In both αβγ-ENaC and δβγ-ENaC assemblies, furin-mediated cleavage of the γ-subunit was observed. Surface-biotinylation experiments further demonstrated that predominantly furin-processed ENaC α- and γ-subunits are present in the plasma membrane, consistent with previous reports on amphibian ENaC orthologues [37]. When extracellular chymotrypsin was applied to oocytes expressing αβγ-ENaC or δβγ-ENaC, an additional cleavage event was observed in the γ-subunit in both ENaC subunit assemblies. This contrasts previous reports on amphibian (*Xenopus laevis*) ENaC orthologues, where the presence of the δ-subunit prevents cleavage of the γ-subunit by chymotrypsin [37]. Recently, Houser and Baconguis (2025) obtained Cryo-EM derived structures of human αβγ-ENaC or δβγ-ENaC assemblies which revealed that the GRIP domain of the γ-subunit rearranges depending on the presence of the neighbouring α- or δ-subunit [15]. Such a rearrangement might prevent chymotrypsin-mediated cleavage of the γ-subunit in *Xenopus* δβγ-ENaC, but not in the guinea pig orthologue. Hence, there are species-specific differences in the mechanisms that prevent or reduce proteolytic ENaC activation in the presence of the δ-subunit.

Furin-mediated cleavage of the α-ENaC subunit removes inhibitory peptides embedded in the P1 strands of the subunit’s GRIP domain and thereby increases channel P_o_ [19, 27]. Consistent with this concept, disruption of the furin consensus sites in the guinea pig α-ENaC subunit strongly reduced amiloride-sensitive currents, thereby confirming that the cleavage state of the α-subunit determines baseline ENaC P_o_. Interestingly, there is an additional arginine-rich motif downstream of the distal furin cleavage site in the guinea pig α-subunit. Functional characterisation of α-ENaC containing a disrupted canonical distal furin cleavage site suggest that this arginine-rich sequence could represent an alternative site for distal furin cleavage which, however, cannot fully replace the canonical distal cleavage site to completely release the inhibitory tract. This could be due to lower affinity or poor accessibility to furin. Whether this observation is only relevant for over-expressed ENaCs in *Xenopus* oocytes or may occur in guinea pigs under physiological conditions in vivo remains to be verified.

Similar to its human counterpart, the guinea pig δ-subunit possesses a markedly shortened GRIP domain and lacks furin consensus sites as well as the P1 inhibitory peptide [11]. The lack of this inhibitory constraint might thereby enhance baseline P_o_ in guinea pig δβγ-ENaC assemblies. Consistent with this hypothesis, experiments employing the β_S521C_-substitution in combination with MTSET treatment, as well as on-cell patch-clamp experiments revealed that the P_o_ of guinea pig δβγ-ENaC is around 90%.

The MTSET experiments suggest a P_o_ for δβ_S521C_γ-ENaC of approx. 94%, assuming that the covalent binding of MTSET to β_C521_ exclusively alters the P_o_ but not the unitary Na^+^ conductance. For rat αβγ-ENaC, it was shown that covalent modification of the analogous β_S518C_ mutant with MTSET has no effect on the unitary Na^+^ conductance [20]. However, the same study showed that MTSET modification of the β_S518C_ mutant reduces unitary Li^+^ conductance by 13% (from 7.8 ± 0.9 pS to 6.8 ± 0.5 pS). Similar results were shown for human αβγ-ENaC, in which the covalent modification of the analogous β_S520C_ mutant leads to a reduction in unitary Li^+^ conductance by 31% (from 8.4 ± 0.21 pS to 5.8 ± 0.23 pS) [31]. However, to our knowledge, the impact of similar MTSET modifications on the unitary conductance of δβγ-ENaCs has not yet been investigated. It is therefore possible that the covalent modification of the β_S521C_ mutant with MTSET reduces the unitary Na^+^ conductance of guinea pig δβγ-ENaC through electrostatic interaction with the permeating Na^+^ ions. Therefore, we may have overestimated the P_o_ of the δβγ-ENaC assembly in the experiments employing the β_S521C_ mutant in combination with MTSET. On the other hand, based on our on-cell patch-clamp recordings, we were able to calculate a P_o_ for δβγ-ENaC of 87.9 ± 20.4%. As explained in the results section, we most likely underestimated the number of δβγ-ENaCs in the membrane patch and, consequently, the P_o_ in these calculations. The true P_o_ of guinea pig δβγ-ENaC is therefore most likely in the range between the values calculated from on-cell patch-clamp and MTSET experiments, i.e. around 90%. This is, to the best of our knowledge, the highest intrinsic P_o_ that has been reported for any ENaC isoform to date.

While furin-mediated removal of the α-subunit’s P1 inhibitory peptide in αβγ-ENaC increases baseline P_o_, maximal activity is achieved by full removal of the P1 inhibitory peptide of the γ-subunit by extracellular proteases [5]. Consistently, chymotrypsin-mediated channel activation was absent in guinea pig αβγ-ENaC assemblies in which the furin consensus site of the γ-subunit was disrupted. Importantly, maximal activation due to proteolytic γ-subunit processing occurred regardless of whether the α-subunit inhibitory tract had been removed, underscoring a mechanistic decoupling between α- and γ-subunit protease-mediated activation pathways [5]. By contrast, neither cleavage of the γ-subunit by chymotrypsin (and trypsin) nor disruption of the furin consensus site in the γ-subunit had any effect on guinea pig δβγ-ENaC activity. This further supports a scenario where guinea pig δβγ-ENaCs are locked in a nearly complete open state and channel activity is therefore uncoupled from proteolytic processing of the γ-subunit.

Aside from the shortened GRIP domain, the guinea pig δ-ENaC subunit has an enlarged knuckle domain due to fusion of exons 11/12 and incorporation of the former intron DNA into the coding sequence of the *SCNN1D* gene [11]. Interestingly, a similar exon fusion has been found in Antilopinae, a subfamily of even-toed ungulates [38], suggesting that this feature evolved at least twice and independently in mammals. Since the δ-subunit knuckle domain can influence the GRIP domain of the adjacent γ-subunit [15], we questioned whether the enlarged knuckle domain might explain the lack of proteolytic-activation of guinea pig δβγ-ENaCs. However, replacing the knuckle domain of the δ-subunit with that of the α-subunit had no effect on baseline ENaC activity and did not alter the lack of response to chymotrypsin. This suggests that the enlarged knuckle domain is not sufficient to explain the high open probability of guinea pig δβγ-ENaC.

In addition to the short GRIP domain and enlarged knuckle, the guinea pig δ-ENaC subunit lacks conserved acidic amino acid residues within the extracellular β6-β7 loop [11], which, in the α-subunit, contribute to a cation binding pocket and facilitate inhibition of ENaC activity by extracellular sodium ions [18, 27, 36]. This mechanism is known as sodium self-inhibition (SSI) and represents a sodium-induced transition of ENaC from a high to a low P_o_ state [34]. While inhibition of furin-mediated processing of the α-subunit enhances SSI, activation of αβγ-ENaC by extracellular proteases decreases SSI. Guinea pig δβγ-ENaC has a lower SSI than αβγ-ENaC [11]. Both the lack of the inhibitory P1 peptide and acidic residues in the β6-β7 loop of the guinea pig δ-ENaC subunit might therefore contribute to the very high baseline P_o_ of guinea pig δβγ-ENaC.

Overall, our data demonstrate that, like human orthologues, control of guinea pig ENaCs by proteases depends on ENaC subunit assembly. While the P_o_ of canonical αβγ-ENaC is dependent on proteolytic cleavage and removal of the α-/γ-subunits’ inhibitory peptides, δβγ-ENaC is locked in an open state and uncoupled from regulation by proteases. Canonical αβγ-ENaCs represent the rate-limiting step for transepithelial sodium (re)absorption in various epithelia such as the renal distal nephron, colon or epithelia lining the respiratory tract [29]. In the lung, the balance between membrane-anchored channel-activating proteases and soluble protease inhibitors adjusts αβγ-ENaC activity to the volume of airway/alveolar lining liquids [24]. By contrast, the physiological function of δβγ-ENaC remains largely unknown [34], but it has been suggested that δβγ-ENaC could function as a sodium sensor, for example, in immune cells [19] or taste buds [4, 38]. The fact that the δβγ-ENaC activity is independent of extracellular sodium concentrations due to a markedly reduced SSI, together with its insensitivity to proteases, would further support such a function. Furthermore, our finding that guinea pig δβγ-ENaC has a P_o_ of around 90% makes this ENaC isoform an interesting candidate for structural studies aiming to reveal the structure of a yet unresolved open state of this important ion channel.

## Supplementary Information

Below is the link to the electronic supplementary material.

Supplementary file1 (PDF 5687 KB)

## Data Availability

Data are made available in the manuscript, and as Source Data deposited at the Zenodo data depository (10.5281/zenodo.18145489).
