# Supplementary material for: Guinea pig δβγ-ENaC is locked in an open state and uncoupled from regulation by proteases
Source: Pflugers Arch. 2026 Apr 25;478(5):43. doi: 10.1007/s00424-026-03173-0 (PMC13109204; doi:10.1007/s00424-026-03173-0)
Supplement: Supplementary file 1 — Supplementary file1 (PDF 5687 KB) [file 424_2026_3173_MOESM1_ESM.pdf]

Supplementary File

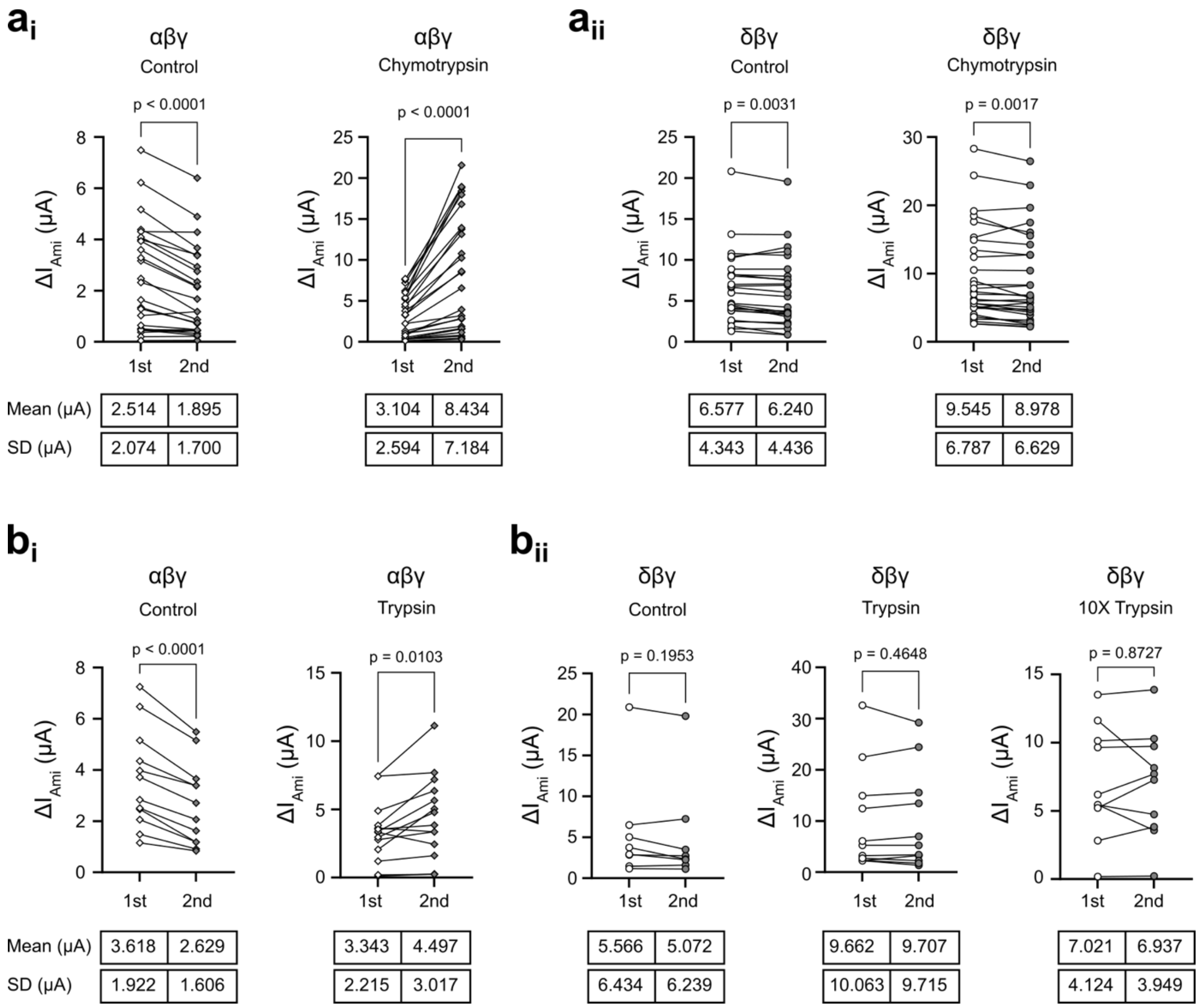

Supplementary Figure 1. Absolute values of ΔI_{ami} before and after protease (or mock treatment) derived from experiments shown in manuscript Figure 1. (a) Shown are ΔI_{ami} of oocytes expressing αβγ-ENaC (**a$_{i}$**) or δβγ-ENaC (**a$_{ii}$**), before (1st) and after (2nd) chymotrypsin (or mock experiments). Statistical analyses were performed using Student's paired t-test or Wilcoxon matched-pairs signed rank test. **(b)** Similar experiments as shown in (a) using trypsin (or mock experiments). Statistical analyses were performed using Student's paired t-test or Wilcoxon matched-pairs signed rank test.

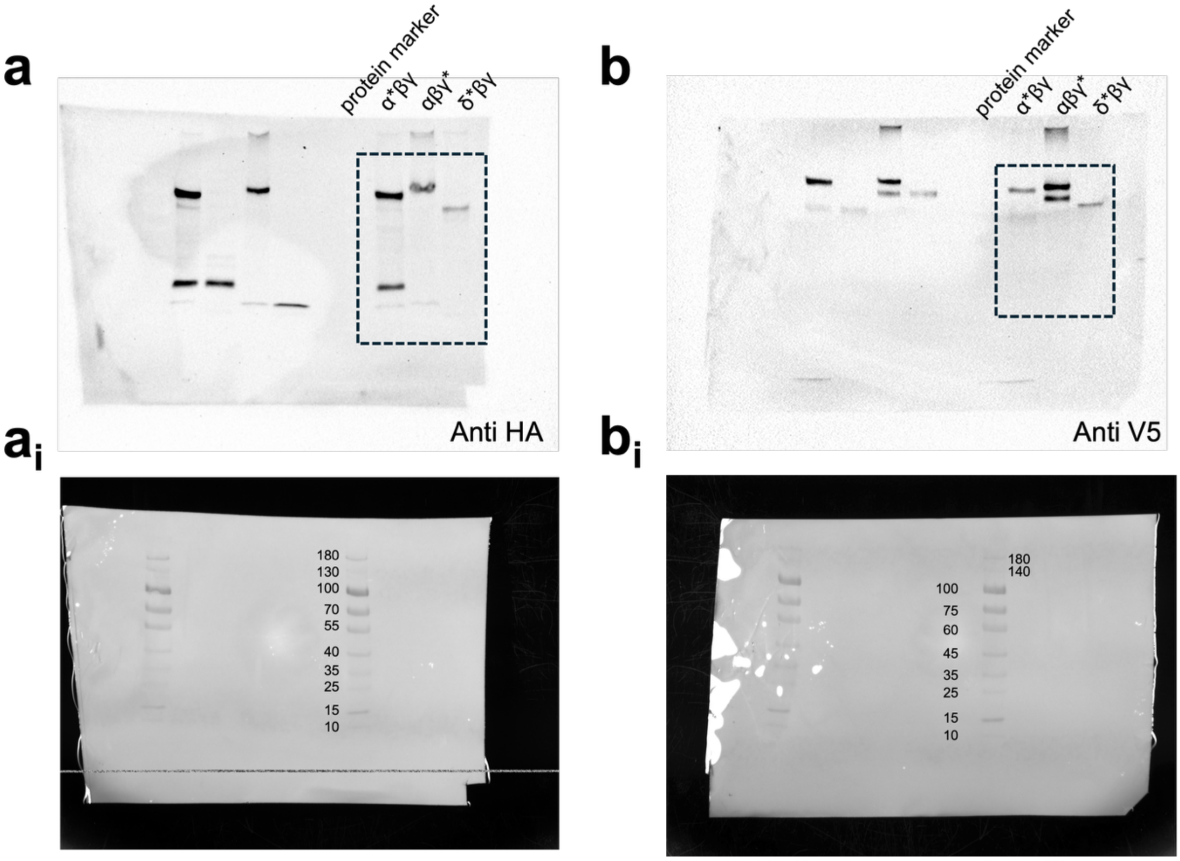

Supplementary Figure 2. Original uncropped versions of the blot images shown in manuscript Figure 2b. Immunoblots using anti-HA (a) and anti-V5 (b) antibodies on whole-cell lysates from oocytes expressing α*βγ-ENaC, αβγ*ENaC, or δ*βγ-ENaC. The blots represent one of n = 2-3 independent experimental repeats. The areas highlighted in dashed boxes indicate the part of the blot shown in manuscript Figure 2b. The additional visible lanes in panel (a) were samples ran on the same gel and used for manuscript Figure 3a (please see Supplementary Figure 2). The additional lanes visible in the image of panel (b) are another replicate of data shown in manuscript Figure 3 b. Panels (a_i) and (b_i) show a photograph of the same blot shown in (a)/(b) to visualise the protein marker (ThermoScientific PageRuler Prestained Protein Ladder for panel for panel a_i; Proteintech Prestained Protein Marker (10-180 kDa) for panel b_i). Numbers indicate protein sizes in kDa.

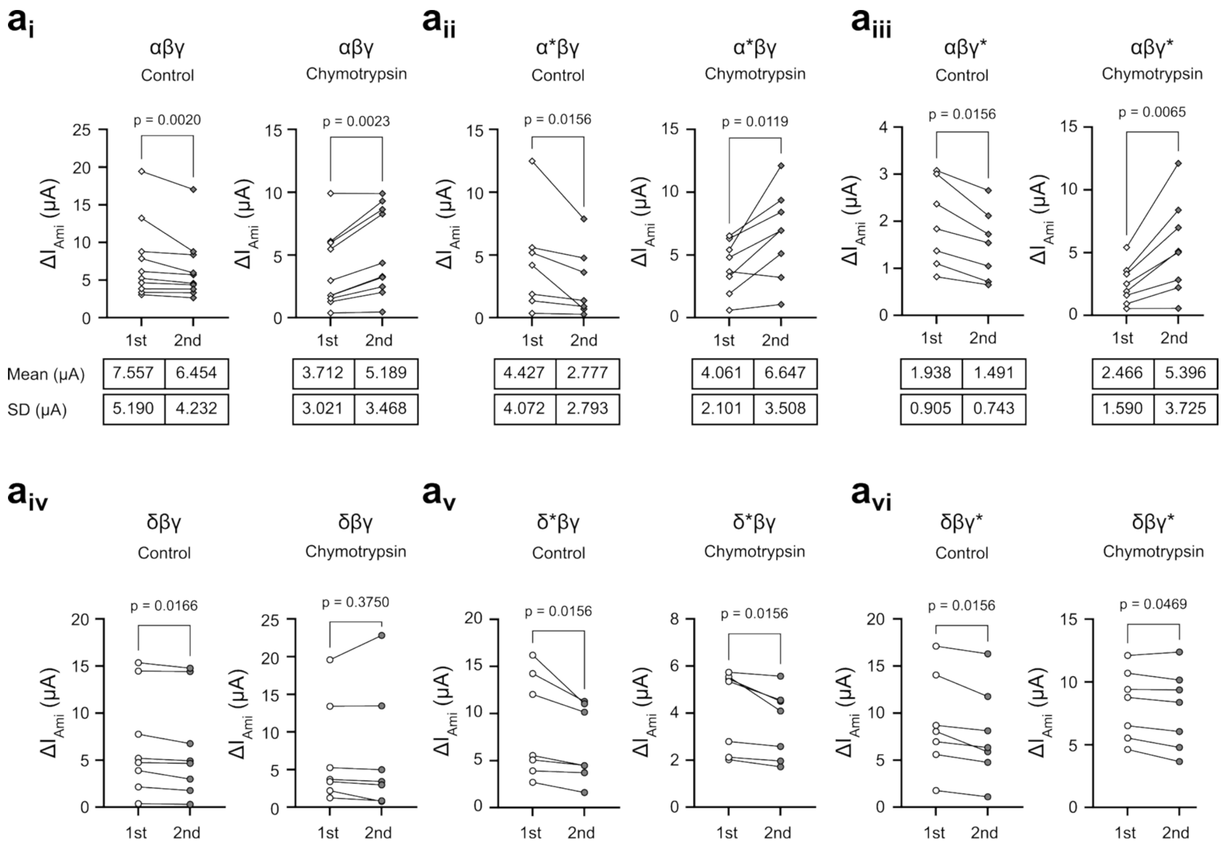

	αβγ Control 1st	αβγ Control 2nd	αβγ Chymotrypsin 1st	αβγ Chymotrypsin 2nd	α*βγ Control 1st	α*βγ Control 2nd	α*βγ Chymotrypsin 1st	α*βγ Chymotrypsin 2nd	αβγ* Control 1st	αβγ* Control 2nd	αβγ* Chymotrypsin 1st	αβγ* Chymotrypsin 2nd
Mean (μA)	7.557	6.454	3.712	5.189	4.427	2.777	4.061	6.647	1.938	1.491	2.466	5.396
SD (μA)	5.190	4.232	3.021	3.468	4.072	2.793	2.101	3.508	0.905	0.743	1.590	3.725

	δβγ Control 1st	δβγ Control 2nd	δβγ Chymotrypsin 1st	δβγ Chymotrypsin 2nd	δ*βγ Control 1st	δ*βγ Control 2nd	δ*βγ Chymotrypsin 1st	δ*βγ Chymotrypsin 2nd	δβγ* Control 1st	δβγ* Control 2nd	δβγ* Chymotrypsin 1st	δβγ* Chymotrypsin 2nd
Mean (μA)	6.739	6.319	6.958	7.029	7.765	5.913	4.144	3.567	8.886	7.749	8.233	7.821
SD (μA)	5.489	5.471	6.862	8.181	5.475	4.013	1.735	1.478	5.165	4.964	2.772	3.126

Supplementary Figure 3. Absolute values of ΔI_{ami} before and after protease (or mock treatment) derived from experiments shown in manuscript Figure 2 d. Shown are ΔI_{ami} of oocytes expressing **(a_i)** αβγ-ENaC, **(a_{ii})** α*βγ-ENaC, **(a_{iii})** αβγ*-ENaC, **(a_{iv})** δβγ-ENaC, **(a_v)** δ*βγ-ENaC or **(a_{vi})** δβγ*-ENaC, before (1st) and after (2nd) chymotrypsin (or mock experiments). Statistical analyses were performed using Student's paired t-test or Wilcoxon matched-pairs signed rank test.

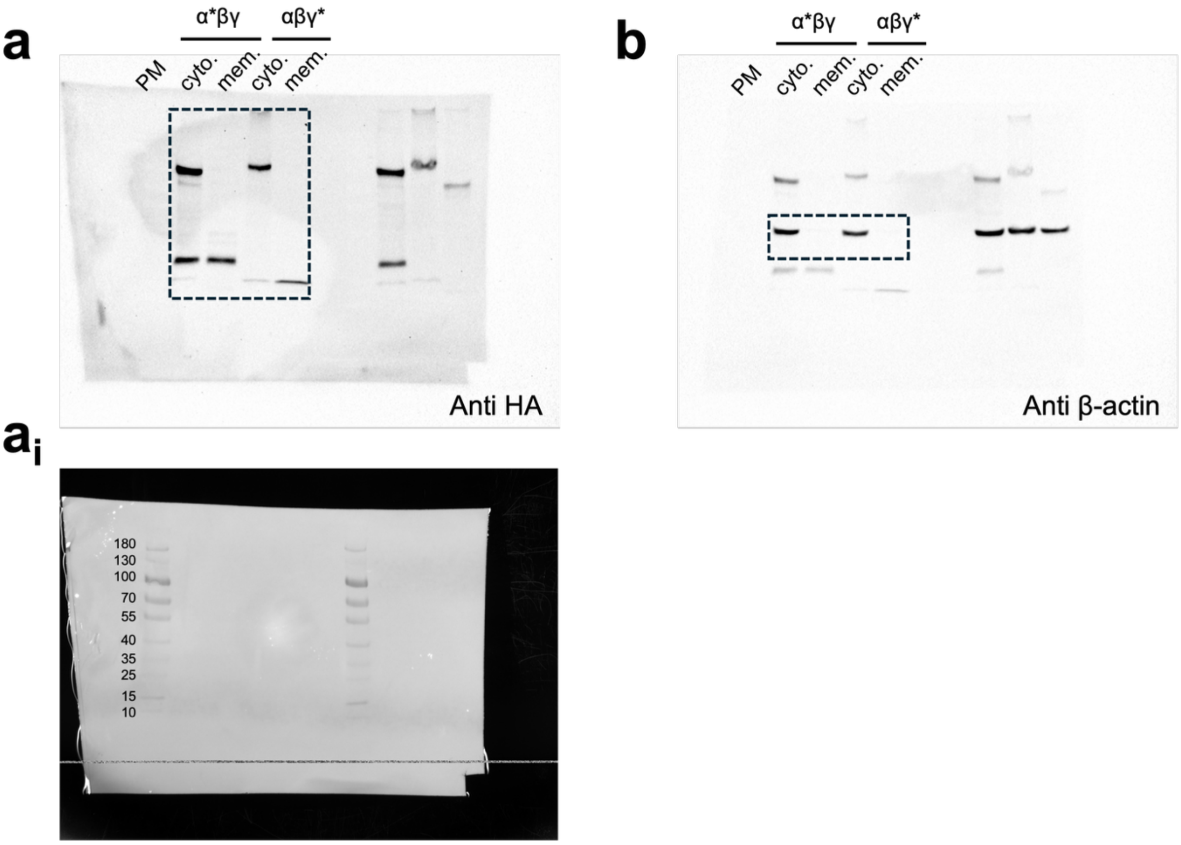

Supplementary Figure 4. Original uncropped versions of the blot images shown in manuscript Figure 3a. Immunoblots following separation of membrane (mem.) from cytosolic (cyto.) fractions of oocytes expressing α*βγ-ENaC, or αβγ*-ENaCs. **(a)** Blots were probed with an anti-HA antibody. **(b)** The same membrane shown in (a) was reprobed with an anti-β-actin antibody to confirm proper separation of membrane from cytosolic fractions. The blots represent one of n = 2-3 independent experimental repeats. The areas highlighted in dashed boxes indicate the part of the blot shown in manuscript Figure 3a. The additional visible lanes were samples ran on the same gel and used for manuscript Figure 2b (please see Supplementary Figure 1). Panel (a_i) shows a photograph of the same blot shown in (a)/(b) to visualise the protein marker (PM; ThermoScientific PageRuler Prestained Protein Ladder). Numbers indicate protein sizes in kDa.

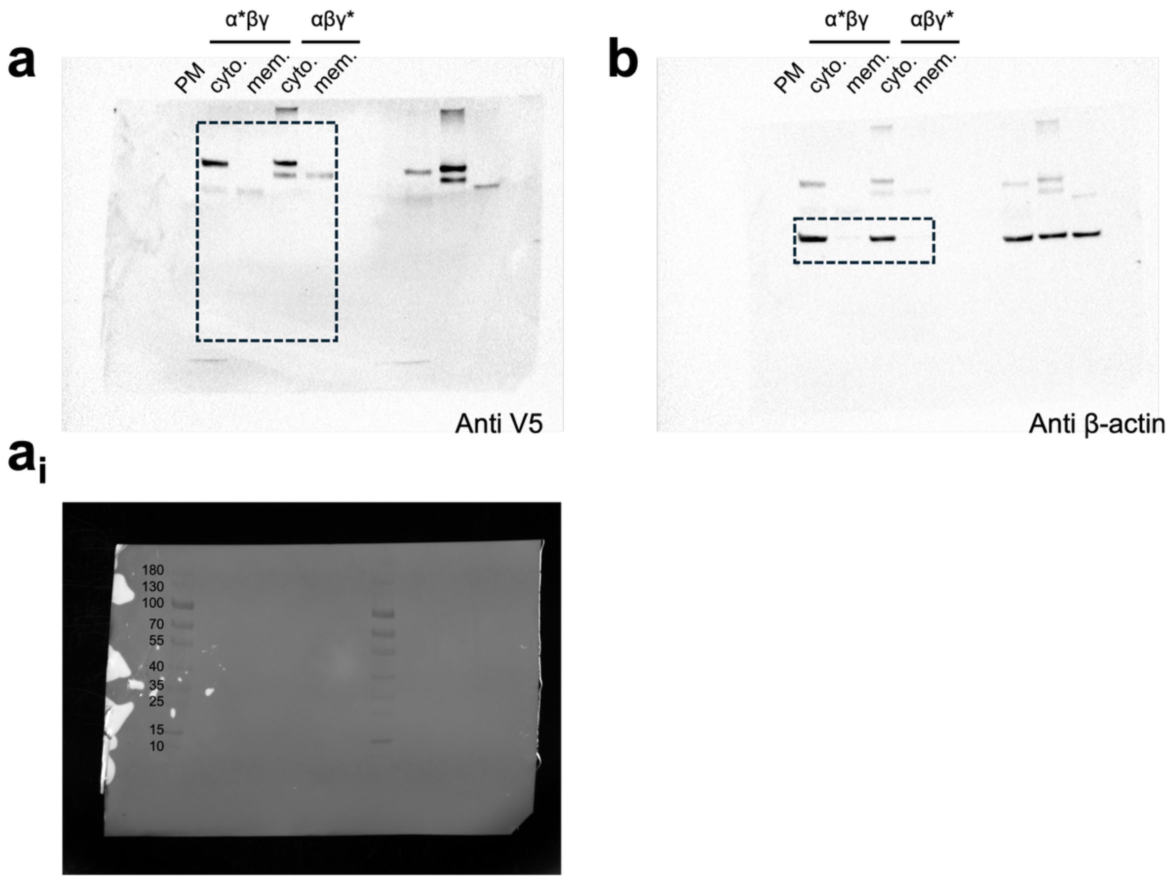

Supplementary Figure 5. Original uncropped versions of the blot images shown in manuscript Figure 3b. Immunoblots following separation of membrane (mem.) from cytosolic (cyto.) fractions of oocytes expressing α*βγ-ENaC, or αβγ*-ENaCs. **(a)** Blots were probed with an anti-V5 antibody. **(b)** The same membrane shown in (a) was reprobed with an anti-β-actin antibody to confirm proper separation of membrane from cytosolic fractions. The blots represent one of n = 2-3 independent experimental repeats. The areas highlighted in dashed boxes indicate the part of the blot shown in manuscript Figure 3b. The additional lanes visible in the image of panel (a) are another replicate of data shown in manuscript Figure 2b. Panel (a_i) shows a photograph of the same blot shown in (a)/(b) to visualise the protein marker (PM; ThermoScientific PageRuler Prestained Protein Ladder). Numbers indicate protein sizes in kDa.

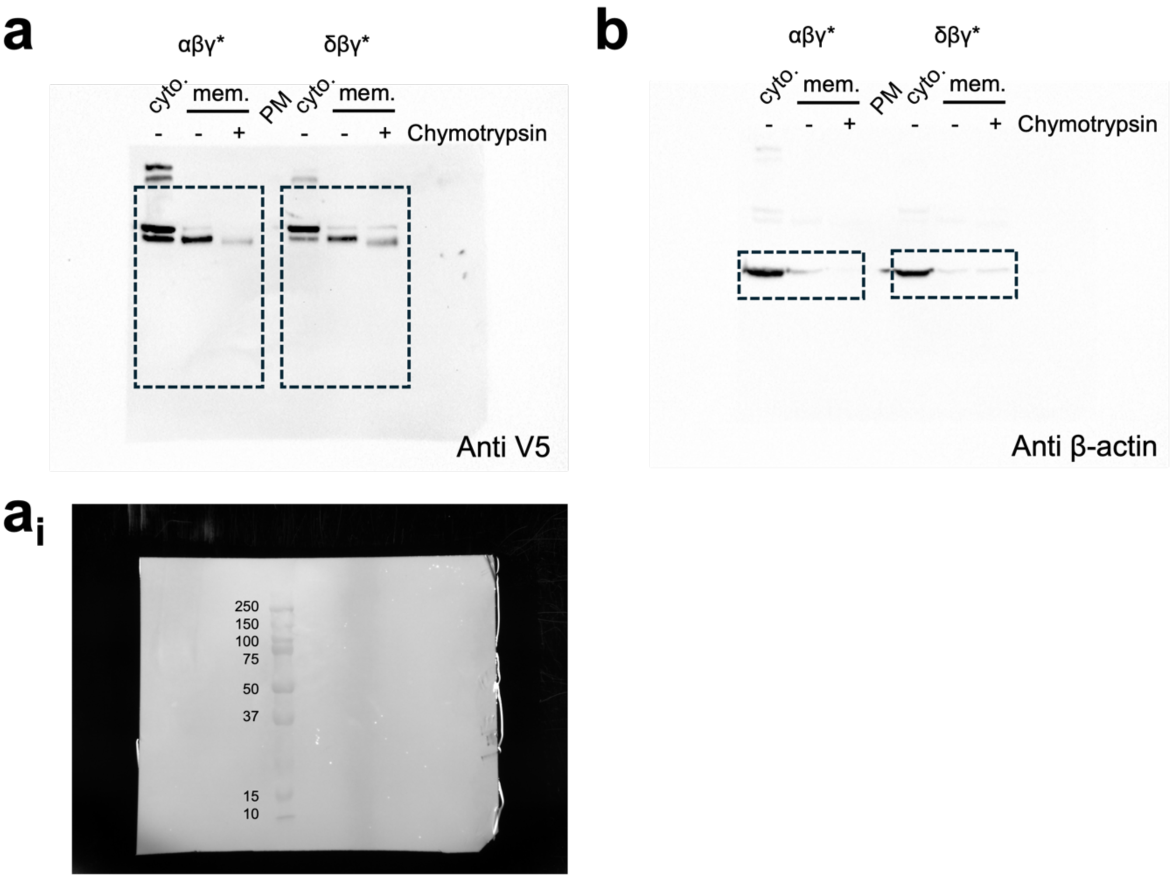

Supplementary Figure 6. Original uncropped versions of the blot images shown in manuscript Figure 3c. (a) To evaluate the ability of extracellular chymotrypsin to cleave the γ-subunit, oocytes were injected with αβγ*-ENaC or δβγ*-ENaC cRNAs. Half the number of oocytes injected per construct were treated with chymotrypsin (2 µg/ml) before biotinylation and immunoblotting using an anti-V5 antibody. Upon chymotrypsin treatment, there was a detectable decrease in the molecular mass of the processed γ*-subunit in both ENaC isoforms. **(b)** Reprobing with anti-β-actin antibody was used to confirm separation of membrane from cytosolic fractions. The blots represent one of n = 3 independent experimental repeats. The areas highlighted in dashed boxes indicate the part of the blot shown in manuscript Figure 3c. Panel (a_i) shows a photograph of the same blot shown in (a)/(b) to visualise the protein marker (PM; Precision Plus Protein Standards, Bio-rad laboratories). Numbers indicate protein sizes in kDa.

a

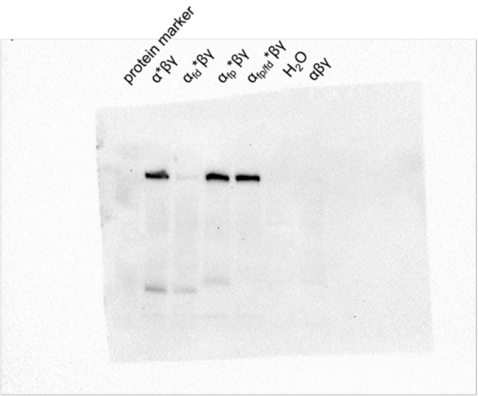

a$_i$

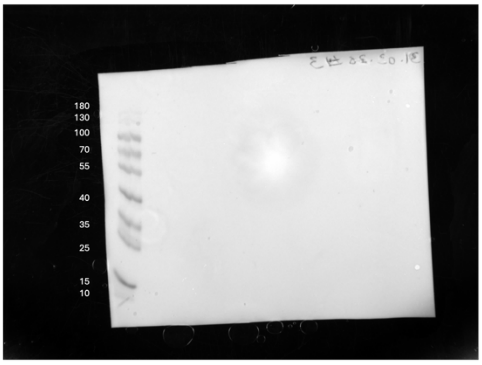

Anti HA

b Guinea pig α*-ENaC

M***YPYDVPDYA***KGDELKAQGPLPPQPLQGPLKGDKCEQPGLGPEPTAPQQHTEEEEALIEFHRSYRELFQFFCNNTTIHGAIRLVCSKHNRMKTAF
WAVLWLCTFGMMYWQFALLFGEYFSYPVSLNINLNSDKLVFPAVTVCTLNPYRYKEIKEQLRELDRITQQTLFDLYNYNASSTLLAGA**RSRR**SLADTL
PYPLQRIPVQPEP**RRAR**SSDPSSVRDNNP**RVDRRDWR**VGFQLCNQNKSDCFYQTSSSGVDGVREWYRFHYINILAQVADTSPSLEEEALGNFIFA
CRFNQAPCTQENYSHFHHPIYGNCYTFNNKNDSSLWMASMPGINNGLSLTLRTEQNDYIPLLSTVTGARVTVHGQDEPAFMDDGGFNLRPGVETS
ISMRKEALDRLGGSYGDCTQDGSDVPVQNLYPSKYTQQVCIHSCFQENMIKQCGCAYIFYPKPKGVEFCDYTNHSAWGYCYYKLQGAFSSDSLGC
FNKCRKPCNVTIYKLSAGYSRWPSAASQDWIFQMLSLQNNYTISNKRNGVAKLNIYFKELNYRTNSESPSVTMVTLLSNLGSQWSLWFGSSVLSVV
EMAEFMFDLLVITLLMLLRRFRSRYWSPGRGARAAREVACTPPPSLPSRFCAHSAFPTLTAPPPAYATLSACPPLQGLAGASSAACAPREP***GKPIPNP***
LLGLDST

Supplementary Figure 7. Original uncropped versions of the blot image shown in manuscript Figure 4b. (a) Immunoblot of whole-cell lysates from oocytes expressing α*βγ-ENaC probed with an anti-HA antibody. The putative furin cleavage sites were sequentially removed, the proximal distal cleavage site (α$_{fd}$*βγ), the proximal furin cleavage site (α$_{fp}$*βγ), and then both sites (α$_{fp/fd}$*βγ). In α$_{fp}$*βγ-ENaC and α$_{fp/fd}$*βγ-ENaC, the band at ~25 kDa disappeared, confirming that cleavage at the proximal site had been impaired. Lysates from oocytes expressing untagged αβγ-ENaC and water-injected oocytes (H_2O) were used to control for unspecific staining. The blot represents one of n = 4 independent experimental repeats (for the tagged ENaC proteins). Panel (a$_i$) shows a photograph of the same blot shown in (a) to visualise the protein marker (ThermoScientific PageRuler Prestained Protein Ladder). Numbers indicate protein sizes in kDa. **(b)** Amino acid sequence of guinea pig α*-ENaC. HA and V5 epitope tags are shown in italic font, the proximal and distal furin sites are indicated as bold and underlined fonts. An ariginine-rich motif downstream of the distal furin site is highlighted in grey.

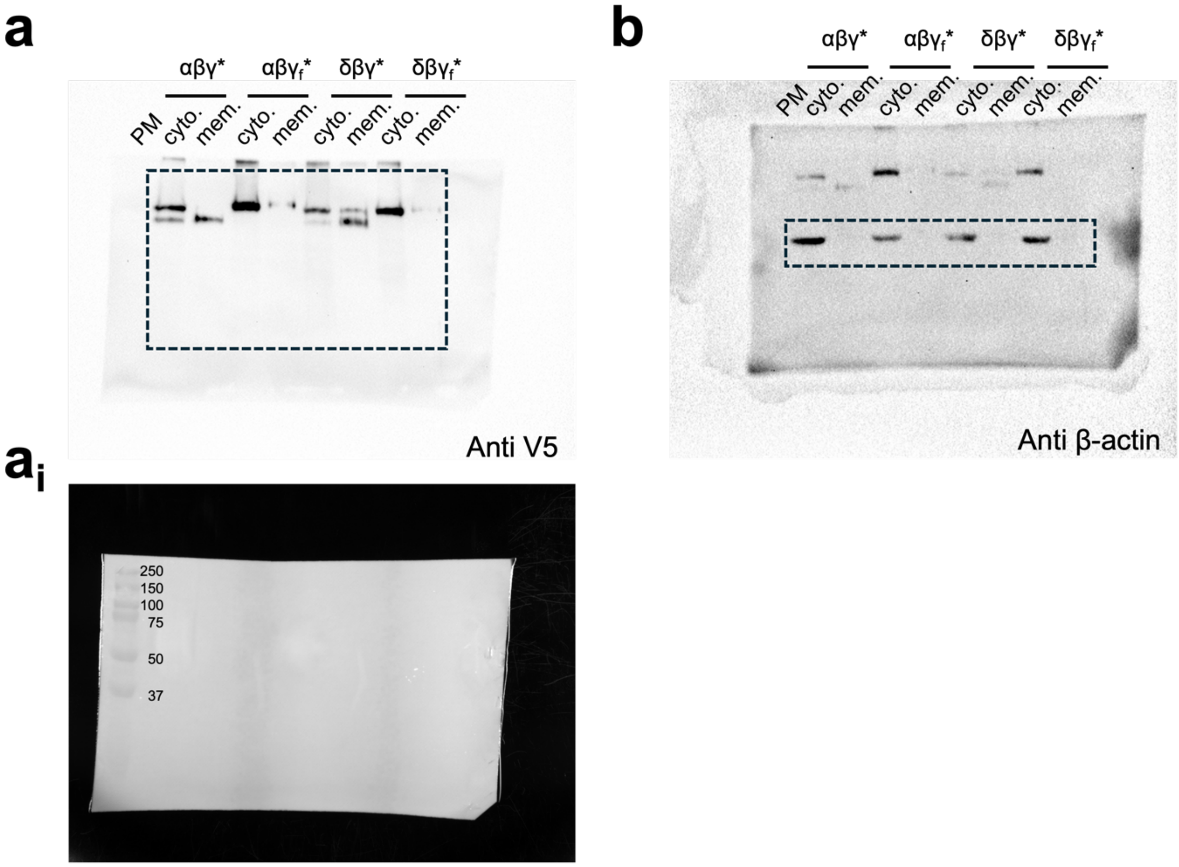

Supplementary Figure 8. Original uncropped versions of the blot image shown in manuscript Figure 4c. (a) Immunoblots on membrane and cytosolic factions derived from oocytes expressing αβγ*-, αβγ_f*-, δβγ*- and δβγ_f*-ENaC probed with an anti-V5 antibody. The cleavage product resulting from furin modification of the γ-subunit disappeared in both αβγ_f*- and δβγ_f*-ENaC assemblies. **(b)** The blot shows the results after reprobing the same membrane shown in (a) with an anti-β-actin antibody to confirm proper separation of membrane from cytosolic fractions. The blot represents one of n = 3 independent experimental repeats. The areas highlighted in dashed boxes indicate the part of the blot shown in manuscript Figure 4c. Panel (a_i) shows a photograph of the same blot shown in (a) to visualise the protein marker (PM; Precision Plus Protein Standards, Bio-rad laboratories). Numbers indicate protein sizes in kDa.

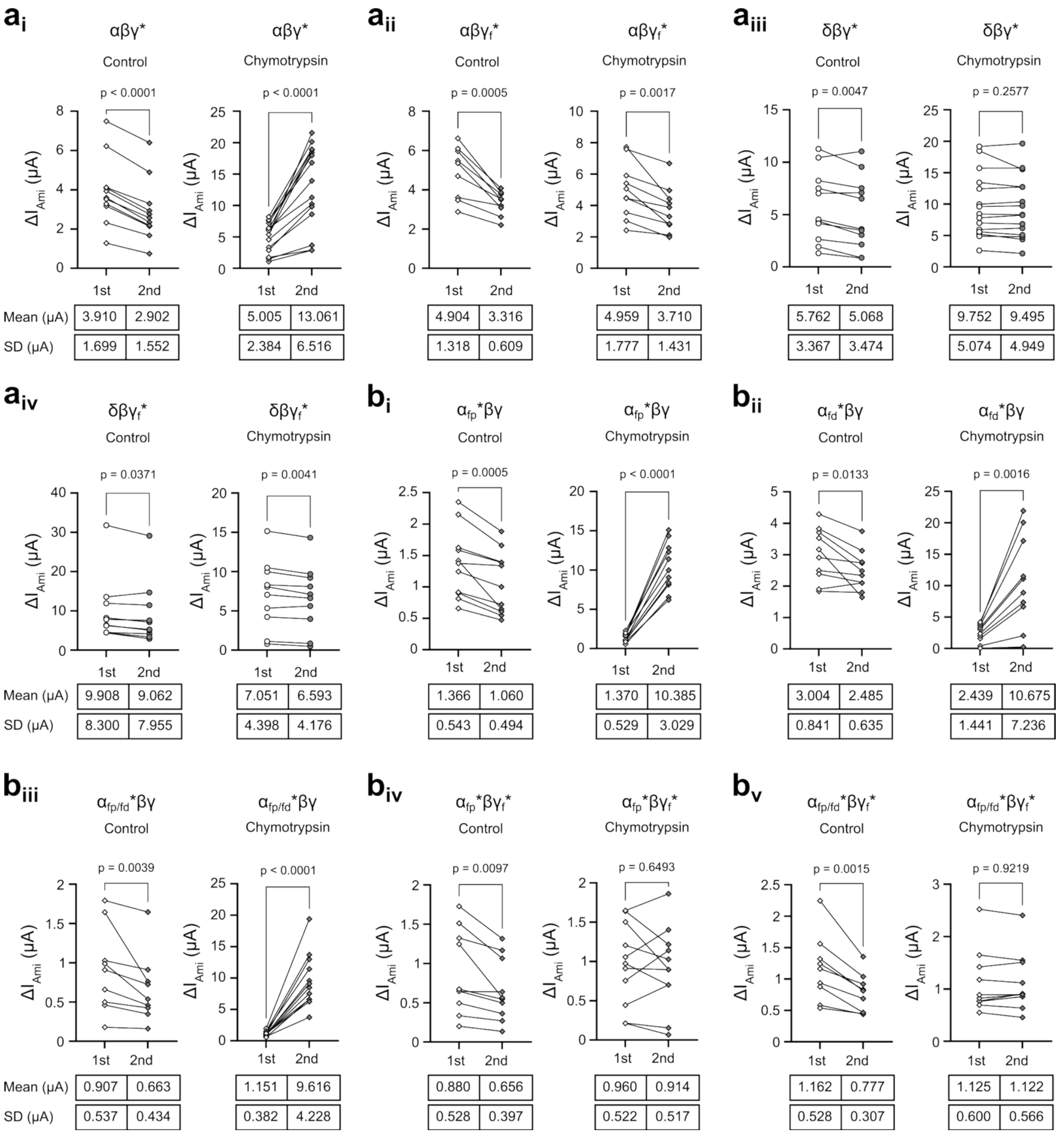

Panel	Condition	Mean (μA) 1st	Mean (μA) 2nd	SD (μA) 1st	SD (μA) 2nd
a_i αβγ*	Control	3.910	2.902	1.699	1.552
a_i αβγ*	Chymotrypsin	5.005	13.061	2.384	6.516
a_{ii} $αβγ_f$*	Control	4.904	3.316	1.318	0.609
a_{ii} $αβγ_f$*	Chymotrypsin	4.959	3.710	1.777	1.431
a_{iii} δβγ*	Control	5.762	5.068	3.367	3.474
a_{iii} δβγ*	Chymotrypsin	9.752	9.495	5.074	4.949
a_{iv} $δβγ_f$*	Control	9.908	9.062	8.300	7.955
a_{iv} $δβγ_f$*	Chymotrypsin	7.051	6.593	4.398	4.176
b_i $α_{fp}$*βγ	Control	1.366	1.060	0.543	0.494
b_i $α_{fp}$*βγ	Chymotrypsin	1.370	10.385	0.529	3.029
b_{ii} $α_{fd}$*βγ	Control	3.004	2.485	0.841	0.635
b_{ii} $α_{fd}$*βγ	Chymotrypsin	2.439	10.675	1.441	7.236
b_{iii} $α_{fp/fd}$*βγ	Control	0.907	0.663	0.537	0.434
b_{iii} $α_{fp/fd}$*βγ	Chymotrypsin	1.151	9.616	0.382	4.228
b_{iv} $α_{fp}$*$βγ_f$*	Control	0.880	0.656	0.528	0.397
b_{iv} $α_{fp}$*$βγ_f$*	Chymotrypsin	0.960	0.914	0.522	0.517
b_v $α_{fp/fd}$*$βγ_f$*	Control	1.162	0.777	0.528	0.307
b_v $α_{fp/fd}$*$βγ_f$*	Chymotrypsin	1.125	1.122	0.600	0.566

Supplementary Figure 9. Absolute values of $ΔI_{ami}$ before and after protease (or mock treatment) derived from experiments shown in manuscript Figure 5. (a) Shown are $ΔI_{ami}$ of oocytes expressing **(a_i)** αβγ*-ENaC, **(a_{ii})** $αβγ_f$*-ENaC, **(a_{iii})** δβγ*-ENaC, **(a_{iv})** $δβγ_f$*-ENaC, before (1st) and after (2nd) chymotrypsin (or mock experiments). Statistical analyses were performed using Student's paired t-test or Wilcoxon matched-pairs signed rank test. **(b)** Shown are $ΔI_{ami}$ of oocytes expressing **(b_i)** $α_{fp}$*βγ-ENaC, **(b_{ii})** $α_{fd}$*βγ-ENaC, **(b_{iii})** $α_{fp/fd}$*βγ-ENaC, **(b_{iv})** $α_{fp}$*$βγ_f$*-ENaC or **(b_v)** $α_{fp/fd}$*$βγ_f$*-ENaC, before (1st) and after (2nd) chymotrypsin (or mock experiments). Statistical analyses were performed using Student's paired t-test or Wilcoxon matched-pairs signed rank test.

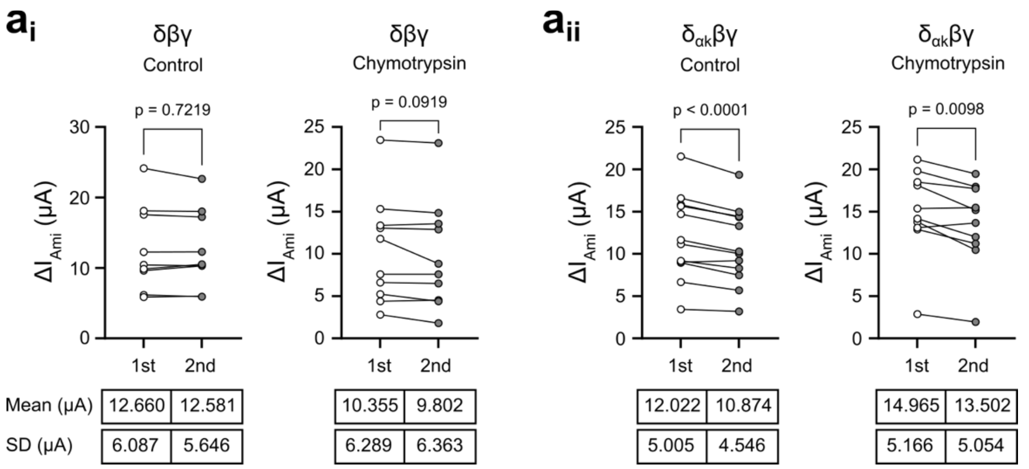

Supplementary Figure 10. Absolute values of ΔI_{ami} before and after protease (or mock treatment) derived from experiments shown in manuscript Figure 6. (Shown are ΔI_{ami} of oocytes expressing **(a_i)** δβγ-ENaC or **(a_{ii})** $\delta_{\alpha k}\beta\gamma$-ENaC, before (1st) and after (2nd) chymotrypsin (or mock experiments). Statistical analyses were performed using Student's paired t-test or Wilcoxon matched-pairs signed rank test.
